# Strategic roadmap to assess forest vulnerability under air pollution and climate change

**DOI:** 10.1111/gcb.16278

**Published:** 2022-06-21

**Authors:** Alessandra De Marco, Pierre Sicard, Zhaozhong Feng, Evgenios Agathokleous, Rocio Alonso, Valda Araminiene, Algirdas Augustatis, Ovidiu Badea, James C. Beasley, Cristina Branquinho, Viktor J. Bruckman, Alessio Collalti, Rakefet David‐Schwartz, Marisa Domingos, Enzai Du, Hector Garcia Gomez, Shoji Hashimoto, Yasutomo Hoshika, Tamara Jakovljevic, Steven McNulty, Elina Oksanen, Yusef Omidi Khaniabadi, Anne‐Katrin Prescher, Costas J. Saitanis, Hiroyuki Sase, Andreas Schmitz, Gabriele Voigt, Makoto Watanabe, Michael D. Wood, Mikhail V. Kozlov, Elena Paoletti

**Affiliations:** ^1^ ENEA, CR Casaccia, SSPT‐PVS Rome Italy; ^2^ ARGANS Biot France; ^3^ Key Laboratory of Agro‐Meteorology of Jiangsu Province, School of Applied Meteorology Nanjing University of Information Science & Technology Nanjing China; ^4^ Ecotoxicology of Air Pollution, CIEMAT Madrid Spain; ^5^ Lithuanian Research Centre for Agriculture and Forestry Kaunas Lithuania; ^6^ Faculty of Forest Sciences and Ecology Vytautas Magnus University Kaunas Lithuania; ^7^ “Marin Drăcea” National Institute for Research and Development in Forestry Voluntari Romania; ^8^ Faculty of Silviculture and Forest Engineering “Transilvania” University Braşov Romania; ^9^ Savannah River Ecology Laboratory and Warnell School of Forestry and Natural Resources University of Georgia Aiken South Carolina USA; ^10^ Centre for Ecology, Evolution and Environmental Changes, Faculdade de Ciências Universidade de Lisboa Lisbon Portugal; ^11^ Commission for Interdisciplinary Ecological Studies Austrian Academy of Sciences Vienna Austria; ^12^ Forest Modeling Lab. ISAFOM‐CNR Perugia Italy; ^13^ Institute of Plant Sciences ARO—Volcani Center Rishon LeTsiyon Israel; ^14^ Instituto de Botanica Nucleo de Pesquisa em Ecologia Sao Paulo Brazil; ^15^ Faculty of Geographical Science Beijing Normal University Beijing China; ^16^ Department of Forest Soils Forestry and Forest Products Research Institute Tsukuba Japan; ^17^ IRET‐CNR Sesto Fiorentino Italy; ^18^ Croatian Forest Research Institute Jastrebarsko Croatia; ^19^ USDA Forest Service Research Triangle Park USA; ^20^ Department of Environmental and Biological Sciences University of Eastern Finland Joensuu Finland; ^21^ Department of Environmental Health Engineering Industrial Medial and Health, Petroleum Industry Health Organization (PIHO) Ahvaz Iran; ^22^ Thuenen Institute of Forest Ecosystems Eberswalde Germany; ^23^ Lab of Ecology and Environmental Science Agricultural University of Athens Athens Greece; ^24^ Ecological Impact Research Department Asia Center for Air Pollution Research (ACAP) Niigata Japan; ^25^ State Agency for Nature, Environment and Consumer Protection of North Rhine‐Westphalia Recklinghausen Germany; ^26^ r.e.m. Consulting Perchtoldsdorf Austria; ^27^ Institute of Agriculture Tokyo University of Agriculture and Technology (TUAT) Fuchu Japan; ^28^ School of Science, Engineering and Environment University of Salford Salford UK; ^29^ Department of Biology University of Turku Turku Finland

**Keywords:** air pollution, climate change, forest ecosystem, forest nutrients, forest research roadmap, forest vulnerability, radioactivity

## Abstract

Although it is an integral part of global change, most of the research addressing the effects of climate change on forests have overlooked the role of environmental pollution. Similarly, most studies investigating the effects of air pollutants on forests have generally neglected the impacts of climate change. We review the current knowledge on combined air pollution and climate change effects on global forest ecosystems and identify several key research priorities as a roadmap for the future. Specifically, we recommend (1) the establishment of much denser array of monitoring sites, particularly in the South Hemisphere; (2) further integration of ground and satellite monitoring; (3) generation of flux‐based standards and critical levels taking into account the sensitivity of dominant forest tree species; (4) long‐term monitoring of N, S, P cycles and base cations deposition together at global scale; (5) intensification of experimental studies, addressing the combined effects of different abiotic factors on forests by assuring a better representation of taxonomic and functional diversity across the ~73,000 tree species on Earth; (6) more experimental focus on phenomics and genomics; (7) improved knowledge on key processes regulating the dynamics of radionuclides in forest systems; and (8) development of models integrating air pollution and climate change data from long‐term monitoring programs.

## Graphical abstract

Air pollution and climate change interact in affecting forest ecosystems. IUFRO experts propose major prospects for protecting vulnerable forests. We need more long‐term ground monitoring sites, satellite monitoring, species‐specific critical levels, combined experimental studies e.g. on phenomics and genomics, understanding of key processes e.g. nutrients cycles and radionuclides dynamics, and integrated modelling.

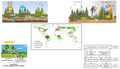

## 1 INTRODUCTION

Forests cover ~30% of the world's land surface, store 45% of terrestrial carbon (Bonan, 2008), and are home to 80% of global terrestrial biodiversity (IUCN, 2021). Sustainable socioeconomic development depends on the proper management of natural resources, including forest ecosystems (Badea et al., 2013). Air pollution and climate change have major impacts on and complex interactions with forest health and productivity (Augustaitis & Bytnerowicz, 2008; Kozlov et al., 2009). For example, tropospheric ozone (O_3_), which is both a phytotoxic gas and a radiative forcer (Myhre et al., 2013), and nitrogen deposition (Du & de Vries, 2018), which causes forest decline due to acidification (Augustaitis et al., 2010) and changes in the frequency and intensity of climatic extremes (e.g., heat waves, rainfall, wind storms), may impact the structure, composition, and functioning of terrestrial ecosystems. These impacts can directly influence carbon cycling and its feedback to the climate system (Frank et al., 2015; Matyssek et al., 2012; Paoletti et al., 2007; Serengil et al., 2011; Sicard et al., 2020).

The future of global forests is a subject of public and political concern due to extensive forest degradation worldwide (Hao et al., 2018; Liu et al., 2018). Recently, environmental pollution was identified as one of the five main drivers of biodiversity loss (European Commission, 2020). Although environmental pollution is an integral part of global change (Dale et al., 2000), most of the research addressing the biotic effects of climate change do not consider this issue. Furthermore, most studies on both the distribution of pollutants and the biotic effects of pollution have neglected the issue of climate change (Sicard, Augustaitis, et al., 2016). As a result, studies exploring the combined effects of air pollution and climate change remain uncommon.

A Web of Science search conducted in June 2021 identified only 74 peer‐reviewed articles containing the keywords “climat* and pollut*” and “tree* or forest*” in the title, 59 of which were relevant research papers (Figure S1): In all, 11 studies used modeling to explore the combined effects of air pollution and climate, 27 studies were based on observations of forest health in either spatial or temporal gradients of air pollution and climate, and only one reported the outcomes of a field experiment. The low number of experimental studies with factorial design involving both airborne pollutants and climate is alarming because it hampers our ability to identify cause‐and‐effect relationships as well as to decipher the mechanisms underlying the combined or interactive effects of pollution and climate on the health of individual trees and forest ecosystems. As a result, the quality of our predictions of the combined effects of climate change and air pollution on future forest health is uncertain. To respond to this global challenge, here we critically review the current knowledge (and gaps) on air pollution and climate interactions in forests, identify key research priorities, and suggest a strategic roadmap for future studies.

## 2 ASSESSING AIR POLLUTION: RESEARCH INFRASTRUCTURES AND METHODOLOGIES FOR FOREST MONITORING

Regional and national air quality directives and emissions control policies (e.g., Japanese Air Pollution Control Act 1968/1970; European Council Directive 2008/50/EC; United States Federal Register, 2015) led to the development of air quality monitoring stations. Monitoring data are collated within national or regional databases, such as the Acid Deposition Monitoring Network in East Asia, the European Environment Agency Airbase system, and the Australia Air Quality Network (AUSAQN; Schultz et al., 2017). Despite efforts to monitor air quality in South America, the spatial distribution of monitoring stations is still heterogeneous and insufficient to represent the pollutant levels (Peláez et al., 2020).

Coordinated research networks of long‐term experimental forest sites integrating monitoring and state‐of‐the‐art methodological and conceptual research to assess air pollution and global change effects are not distributed in a way that represents all forest ecosystem types over the globe. Long‐term forest monitoring and infrastructure networks are running regionally and worldwide, even overlapping each other in their geographic expansions, and are likely to further expand in the future. Here, we introduce some of the largest networks of experimental forest sites, their research aims and methodologies, and explore their capacities in view of the *Supersite* definition (Mikkelsen et al., 2013).

International Long‐Term Ecological Research (ILTER) is a “network of networks” with research sites located in a wide array of ecosystems aimed at developing a global understanding of environmental change while also covering socioeconomical aspects (known as LTSER). Expertise warrants the collection, management, and analysis of spatiotemporally diverse datasets, such as DEIMS (Drupal Ecological Information Management System), a central platform providing information on sites and networks with ecological long‐term monitoring and experimentation at European and global scales. Currently, ILTER encompasses 39 countries which together operate more than 600 sites (Maass et al., 2016). Some sites maintain advanced continuous measurements, such as tower‐based eddy covariance assessments of CO_2_ and H_2_O fluxes. The ILTER network includes the Terrestrial Ecosystem Research Network (TERN), established in Australia, which provides a comprehensive metadata portal containing information on continental scale datasets of measurements describing fauna, flora, terrestrial ecosystems, ecological dynamics, land surfaces, soils, agricultural ecosystems, coasts, climate observations and fluxes (Karan et al., 2016). Similarly, the Chinese National Ecosystem Research Network (CNERN) is an integrated platform of field stations supervised by various Chinese ministries. CNERN represents a science and technology system that conducts network observation and experimentation across China's ecosystems, cutting across governmental departments, industrial sectors, regions, and jurisdictions, and seeks to integrate observation equipment and data resources and standardize research methods, tools, and protocols. As a result, CNERN serves as a nexus for national ecological research, promotes data sharing, and creates an educational center and collaborative base for ecological research. ILTER networks are also present in Korea and Taiwan.

Another “network of regional networks” is represented by FLUXNET, which is coordinating regional and global analyses conducted at micrometeorological tower sites (eddy covariance) to investigate the exchanges of carbon dioxide (CO_2_), water vapor, and energy between terrestrial ecosystems and the atmosphere (Pastorello et al., 2020). FLUXNET is divided into regional networks, for example, the European Integrated Carbon Observation System Research Infrastructure (ICOS RI) with more than 100 measuring stations including 32 forest stations. In 2021, more than 800 sites worldwide were operated on a long‐term and continuous basis within this network. Habitats included in this monitoring framework include temperate conifer and broadleaf (deciduous and evergreen) forests, tropical and boreal forests, crops, grasslands, chaparral, wetlands, and tundra.

In Europe, the International Co‐operative Programme on Assessment and Monitoring of Air Pollution Effects was launched in 1985 under the United Nations Convention on Long‐Range Transboundary Air Pollution (CLRTAP), with several units including ICP Forests (Michel et al., 2018), ICP Vegetation, ICP Modelling and Mapping, and ICP Integrated Monitoring (Lundin & Forsius, 2004). Networks of monitoring stations are established within this framework that continuously assess ecosystem responses to air pollution and develop the associated modeling and assessment methods (Forsius et al., 2021). ICP Forests currently monitor forest conditions in Europe at two intensities: Level I is based on around 6000 observation plots within a systematic transnational grid of 16 × 16 km^2^. Level II comprises around 800 plots in selected forest ecosystems for clarifying cause–effect relationships, and also assesses foliar and soil chemistry, tree growth, and conditions of ground vegetation. Approximately 41 sites, depending on the parameters, also monitor ambient air quality and meteorology.

**FIGURE 1:**
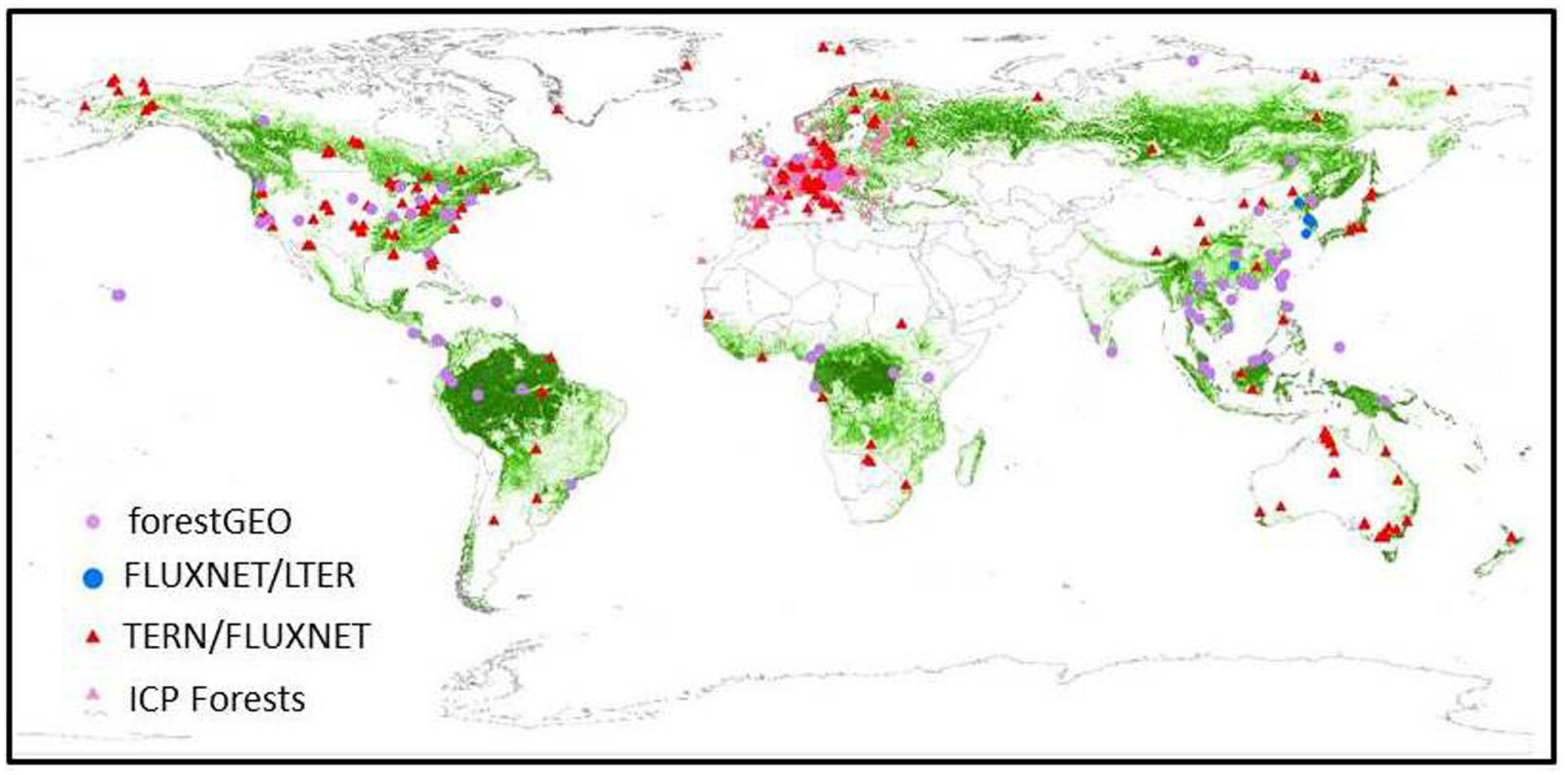
Distribution of the most relevant monitoring network over the forested areas of the globe.

ForestGEO is a global network of scientists and forest research sites dedicated to advancing long‐term study of the world's forests, dedicated to the study of tropical and temperate forest function and diversity. The multi‐institutional network comprises 73 forest research sites across the Americas, Africa, Asia, Europe, and Oceania. ForestGEO monitors the growth and survival of approximately 6 million trees and nearly 13,000 species that occur in the forest research sites. This network also supports initiatives to monitor attributes such as climate, carbon flux, vertebrates, insects, and soil microorganisms. ForestGEO increases scientific understanding about the potential effects of climate change on ecosystems, which is a priority of the US Climate Change Science Program and highlighted by the Intergovernmental Panel on Climate Change (IPCC) Working Group II. Because of ForestGEO's extensive biological monitoring, unique databases, and the partners' expertise, it promises to enhance society's ability to evaluate and respond to the impacts of global climate change. To date, unfortunately, the distribution of forest monitoring sites within ForestGEO appears non‐homogeneous (Figure 1). Indeed, boreal and tropical forests are less represented among monitoring sites and there is a disproportionate number of monitoring sites in the Northern Hemisphere (NH), particularly in Europe, and fewer sites in the Southern Hemisphere (SH).

The aforementioned monitoring networks may benefit from data derived through remote sensing measurements (Lechner et al., 2020). Remotely sensed imagery provides a synoptic view, and is potentially available everywhere at a large range of spatial and temporal scales with a high degree of homogeneity. Furthermore, remote imagery provides digital images that can easily be integrated with other spatial datasets in a geographic information system, and per unit area remote sensing is an inexpensive way to acquire data. The most used remote sensing sensors for assessing and monitoring forest conditions are those on‐board satellites, followed by airborne (including Unmanned Aerial Vehicles) and terrestrial systems, or a combination of these platforms (Torres et al., 2021). Previous studies have demonstrated the utility of optical remote sensing for assessing a variety of forest health indices, and are commonly used in forest monitoring activities (Curran et al., 1992; Huang et al., 2019; Parent & Verbyla, 2010). Landsat satellite images are still the most widely used Earth Observation (EO) data in forest health studies (Torres et al., 2021), which provide continuous time series data from the 1970s (i.e., Landsat 1 mission) until today (i.e., Landsat 8). Access to Landsat images has been free since 2008, and the recently launched Landsat 9 (September 2021) will be publicly available in early 2022.

In addition to Landsat imagery, imagery from sentinel missions from the European Space Agency is also particularly important for forest monitoring because of their high spatial and temporal resolution. Furthermore, the availability of both active (Sentinel‐1) and passive (Sentinel‐2) sensors might increase the precision of previous analytical methods that rely primarily on optical reflectance indices. Similarly, forest health monitoring studies are increasingly using Synthetic Aperture Radar (SAR) sensors. For example, C‐band data are sensitive to variations of Leaf Area Index, which are connected to defoliation and hence forest status (Manninen et al., 2003; Stankevich et al., 2017). SAR sensors are advantageous not just because of their sensitivity to forest structural changes (Dobson et al., 1992; Harrell et al., 1995; Le Toan et al., 1992), but also because of their ability to monitor the water content of the tree canopy (Dobson et al., 1992; Harrell et al., 1995; Le Toan et al., 1992).

Specific remote sensing techniques that merge different spatial, spectral, radiometric, and temporal resolutions are being increasingly used to reduce data gaps and to characterize forest ecosystems (Lausch et al., 2018). For example, Rogers et al. (2018) demonstrated the potential of derived products based on Landsat, Advanced Very High‐Resolution Radiometer (AVHRR), and MODIS (Moderate Resolution Imaging Spectroradiometer) data to detect early signals of tree mortality. Modeling various biophysical indicators based on aerial or ground‐based LiDAR data can further expand the portfolio of remote sensing‐derived data, or at the very least allow their validation in a more efficient manner than by means of traditional monitoring and inventory. In this regard, a fusion of satellite spectral data (e.g., Sentinel‐2) and LiDAR data (Global Ecosystem Dynamics Investigations) could be the next step for global drought‐induced tree mortality assessment (Huang et al., 2019). More recently, the Copernicus air‐pollution monitoring satellite dedicated to trace gasses assessment, such as O_3_, NO_2_, SO_2_, formaldehyde (HCHO), CO, and CH_4_ (Sentinel‐5—Precursor/TROPOMI; Inness et al., 2019), has been used for tracking pollution events and pollution sources (Mesas‐Carrascosa et al., 2020). By merging classical monitoring techniques and state‐of‐the‐art remote sensing, long‐term studies are facilitated (Tănase et al., 2019). Remote sensing use should be expanded to vulnerable regions or ecosystem types which need special protection from climate change and air pollution.

Highly instrumented forest research infrastructures (supersites) provide long‐term data series and promote integration of research communities in a transcontinental collaboration network (Fischer et al., 2011). For these supersites, the use of forest inventory data together with remote sensing and EO data can provide valuable information on forest condition (Hartmann et al., 2018). As new forest change detection algorithms based on EO sensors are developed, they can be validated using data from long‐term monitoring networks (Rodman et al., 2021).

To understand climate change and weather extremes, it is important to have observations of the Earth system going back as far as possible in time. Reanalysis combines past short‐range weather forecasts with observations through data assimilation (Uppala et al., 2005). The process mimics the production of day‐to‐day weather forecasts. Reanalyses are usually produced at lower resolution than current weather forecasts, and they use the same modern‐data assimilation system and forecasting model throughout the reanalysis period. The latest European Centre for Medium‐Range Weather Forecasts (ECMWF) reanalyses are produced through the EU‐funded Copernicus Climate Change Service (C3S). Forecasts are freely available through the C3S Climate Data Store. The most recent ECMWF reanalysis dataset is the ERA5 Back Extension, providing data from 1950 to 1978. The Copernicus Atmosphere Monitoring Service (CAMS) provides continuous data and information on atmospheric composition. The service describes the current situation, forecasts the situation a few days ahead, and analyses consistently retrospective data records for recent years. CAMS supports many applications in a variety of domains including health, environmental monitoring, renewable energies, meteorology, and climatology. CAMS monitors and forecasts European air quality and worldwide long‐range transport of pollutants.

## 3 ELEMENT DEPOSITION IN GLOBAL FORESTS

**FIGURE 2:**
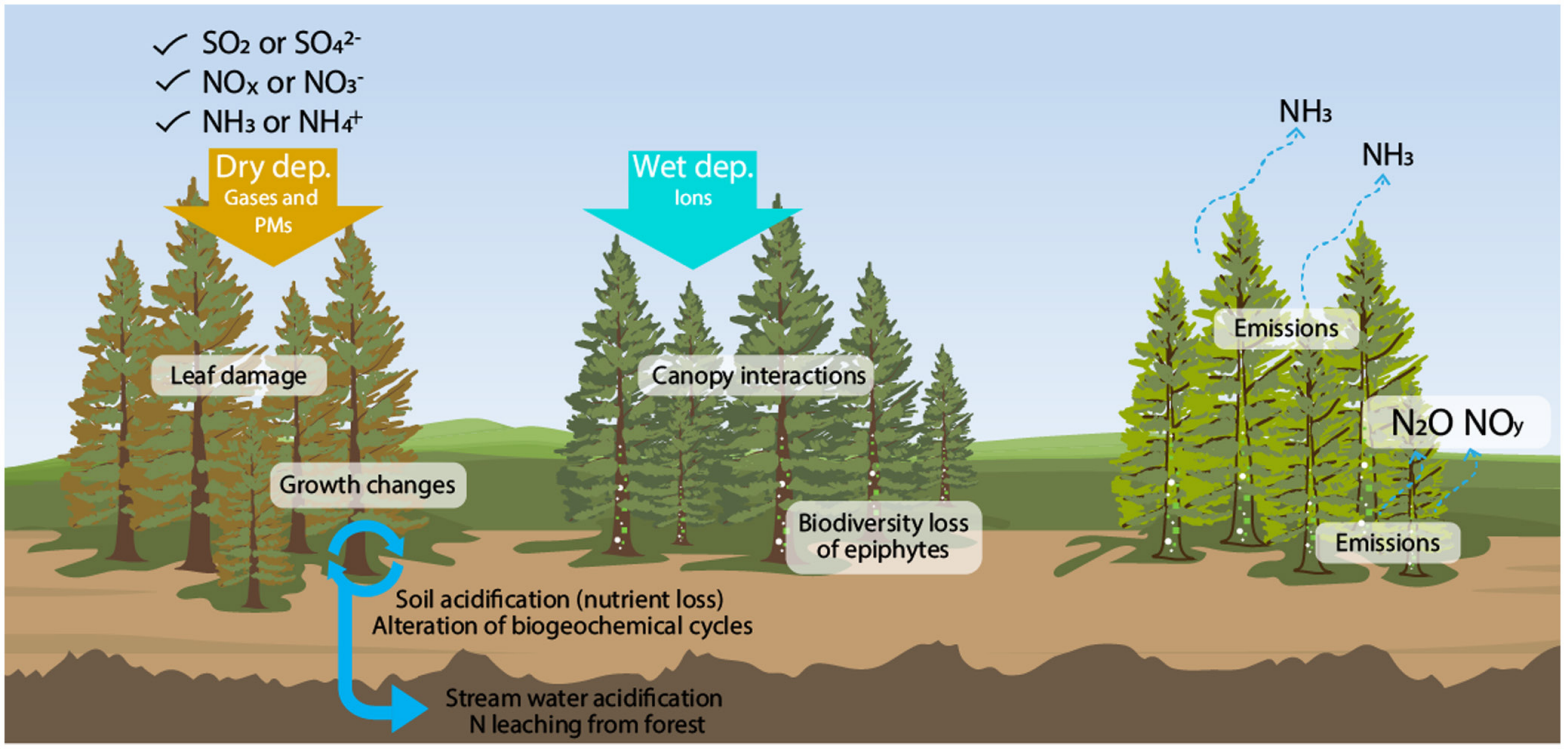
Main interactions of forest ecosystems with sulfur (S) and nitrogen (N) compounds. They may function as either stressors (S) or nutrients (N), even when they are derived from anthropogenic air pollutants, such as sulfur oxides (SOx), nitrogen oxides (NOx), and ammonia (NH_3_), with direct effects on forest canopy (Du et al., 2017) and indirect effects on acidification (Augustaitis et al., 2010) and eutrophication (de Vries, 2021) including impacts on biodiversity (Clark et al., 2013), growth (Du et al., 2018), volatile emissions (Hansen et al., 2017; Liu & Greaver, 2009; Mushinski et al., 2019; Schindler et al., 2020; Xie et al., 2018), and biogeochemistry (Gaudio et al., 2015; Nakahara et al., 2010).

Various substances emitted from natural or anthropogenic sources flow from the atmosphere into forest ecosystems by either wet or dry deposition (Tørseth et al., 2012). Atmospheric deposition may be harmful or beneficial for trees and other plants (Figure 2). Sulfur (S) and nitrogen (N) compounds may function as either nutrients or stressors for forests, even though they are derived from anthropogenic air pollutants, such as sulfur oxides (SO_x_), nitrogen oxides (NO_x_), and ammonia (NH_3_; Duan et al., 2016; Oksanen & Kontunen‐Soppela, 2021). When traveling through the canopy, acid deposition can cause direct damage to plant leaves (Du et al., 2017). When deposited to the forest floor, N and S compounds are identified as a cause of acidification and eutrophication (or N saturation) of forest ecosystems (de Vries, 2021). Moreover, certain amounts of phosphorus (P) and basic cations, such as calcium (Ca^2+^) and magnesium (Mg^2+^), acting in forests as nutrients, are also derived from anthropogenic emissions (Du et al., 2016, 2018). Climate change may directly or indirectly affect the roles of these substances in forest ecosystems (e.g., Mitchell & Likens, 2011; Nakahara et al., 2010).

Atmospheric deposition, especially of S and N compounds, has declined over the last three decades (Sicard, De Marco, et al., 2016; Tørseth et al., 2012; Zhong et al., 2020), despite many developing nations still lacking effective SO_2_ emission controls. In Europe, deposition of S and N peaked in the late 1970s and in the 1980s, respectively (Engardt et al., 2017). In North America, deposition of S and N peaked in the early 1970s and mid‐1990s, respectively (Mitchell & Likens, 2011), when NH_3_ emission became more important (Du, 2016). In South America, most average daily concentrations of SO_2_ are below the World Health Organization air quality guidelines (Peláez et al., 2020), and global atmospheric S deposition is lower than in Europe, Asia, the United States, and Africa (Gao et al., 2018), ranging around 4.96 ± 3.45 kgS ha^−1^ a^−1^. In Asia, emissions of SO_2_ and NO_x_ significantly increased from the early 1980s to the early 2000s (Ohara et al., 2007), 20 or 30 years later than in Europe and the United States. The emissions of SO_2_ and NO_x_ in China peaked in 2006 (Lu et al., 2011) and 2011–2012 (Zheng et al., 2018), respectively, and thereafter started decreasing. In China, emissions of NH_3_ reached a plateau in 1996 (Kang et al., 2016), although a gradual increase in NH_3_ emissions in Asia (including China) was observed as of 2015 (Kurokawa & Ohara, 2020). A recent global analysis combined inventory and modeling data to confirm that the total annual NO_x_ emissions finally stopped increasing in 2013, largely due to strict control measures taken in China in recent years (Huang et al., 2017). However, SO_2_ emissions in India overtook those in China in 2016 (Li et al., 2017), and thus a focus should be placed on monitoring atmospheric deposition in India and other developing countries. Major air pollutants have been changing with industrialization in each region, from SO_2_ to NO_X_ and NH_3_. With temporal changes of major pollutants relative to industrialization, acidification, photochemical formation of ozone, and excess N deposition appeared in sequence as problems for forest ecosystems, as seen in the changes of main causes of tree and forest decline in Northeast Asia (Takahashi et al., 2020). Thus, emission reduction of S and/or N has been reflected gradually by the conditions of forest ecosystems.

In Europe and the United States, reduced S deposition resulted in long‐term declines in ${\mathrm{SO}}_{4}^{2-}$ concentrations in soil solutions (Berger et al., 2016; Johnson et al., 2018) and freshwater (Garmo et al., 2014; Vuorenmaa et al., 2017). However, since S compounds are retained in forest ecosystems and released with changing environmental conditions, changes in S leaching do not necessarily occur at the same time as S deposition changes. Therefore, S output in forest catchments often exceeds the atmospheric input due to legacy S pools derived from past deposition (Vuorenmaa et al., 2017) or due to changing climate (Mitchell & Likens, 2011), which might delay recovery from acidification. In Asia, much of the deposited atmospheric ${\mathrm{SO}}_{4}^{2-}$ seems to be retained in forest soils (Duan et al., 2016; Sase et al., 2019), which may imply a future risk of soil acidification under changing climate. In fact, ${\mathrm{SO}}_{4}^{2-}$ concentrations and pH of river waters are related to the S emission/deposition rate (Duan et al., 2011; Qiao et al., 2016; Sase et al., 2017, 2021). To understand the S cycle in forest ecosystems, targeted studies on deposition trend and changing climate are required (e.g., Mitchell & Likens, 2011; Sase et al., 2019, 2021; Vuorenmaa et al., 2017).

Air pollution abatement may also reduce atmospheric inputs of base cations (Tørseth et al., 2012), as reported for forest soil solutions (Johnson et al., 2018) and freshwaters (Garmo et al., 2014; Stoddard et al., 1999). Base cation nutrients in China forests neutralized on average 76% of the potential acid load due to acid deposition during 2001–2015 (Du et al., 2018). Thus, base cation deposition should be monitored simultaneously along with S and N deposition as already done by several networks globally to assess nutrient status and recovery from acidification in forest ecosystems.

Excess N inputs from the atmosphere have been disturbing biogeochemical cycles in forest ecosystems (e.g., Aber et al., 1989; Nakahara et al., 2010). With reduction in total N deposition mainly due to NO_X_ emissions, an improvement is expected in the NH. However, high levels of NH_3_ deposition are still concerning because NH_3_ emissions have not clearly reduced in many of the regions as described above. Moreover, since emissions of SO_2_ and NO_x_ have been reduced resulting in significant decline of particulate formation (such as (NH_4_)_2_SO4 and NH_4_NO_3_), air concentrations of NH_3_ have been increasing and accordingly more localized NH_3_ deposition was identified in the United States (Butler et al., 2016). Even though regional N deposition has gradually decreased, ecosystem responses to N deposition appeared to show some degree of hysteresis (Gilliam et al., 2019). In fact, there was no large‐scale response in understory vegetation, tree growth, or vitality to reduction of N deposition in Europe, while a decline in ${\mathrm{NO}}_{3}^{-}$ concentrations in soil solutions and foliar N concentrations were partly observed (Schmitz et al., 2019). In Asia, three decades of increase in N deposition in China have exerted significant impacts on soil and water acidification, understory biodiversity, forest growth, and carbon sequestration (Qiao et al., 2016; Tian et al., 2018). However, recovery from acidification and N saturation has already started following a reduction in N deposition in Japan (Sase et al., 2019), where high S and N deposition and climatic anomalies caused acidification and N saturation in the 1990s (Nakahara et al., 2010). Nitrogen leaching from forest ecosystems is controlled not only by N deposition, but also by various factors, including tree age, forest management, climate, and other limiting nutrients such as phosphorus. Moreover, emissions of NH_3_ (e.g., Hansen et al., 2017), N_2_O (e.g., Schindler et al., 2020; Xie et al., 2018), and NOy (as NO + NO_2_ + HONO; e.g., Mushinski et al., 2019) as well as microbial nitrification rate (e.g., Fang et al., 2015) in forest areas should be taken into consideration for actual N fluxes. Since N deposition may increase gas N emissions from ecosystems (e.g., Xie et al., 2018), a comprehensive study considering bilateral N fluxes (both deposition and emission) should be promoted to evaluate whether a forest ecosystem is a sink or source of reactive N species.

The analysis of N dynamics in Latin America is complex, due to the enormous diversity of unmanaged and managed ecosystems, including arid deserts as well as temperate and tropical forests. Cunha‐Zeri and Ometto (2021) stated the major input of N in Latin American countries over the past decades occurred via natural biological fixation, compared to anthropic sources (fertilizers and fossil fuel combustion). Nevertheless, human activities have currently changed the N cycle of natural ecosystems in Latin America. For instance, the conversion of unmanaged land to agriculture increased biological N fixation up to twofold (Reis et al., 2020). Although the highest total N deposition occurs in eastern and southern China, Japan, Eastern US, and European forests, the highest dry deposition occurs in tropical forests (Schwede et al., 2018). For instance, dry N deposition into the Atlantic Forest in the city of São Paulo (Brazil) can exceed the critical N load found for most forests (Souza et al., 2020).

Because of the continued increase in NH_3_ emission in some regions (e.g., Kurokawa & Ohara, 2020) and stagnating values in others (Maas & Grennfelt, 2016), N deposition is a pervasive issue that impacts forest ecosystems. In addition, even relatively low levels of N deposition affect the mycorrhizal association of trees (Lilleskov et al., 2019; van der Linde et al., 2018) and may affect biodiversity of sensitive species, such as lichens (Giordani et al., 2014). The magnitude and consequences of these human‐induced changes in plant–soil–microbe interactions as well as potential pathways for recovery are currently open questions.

Moreover, excess N deposition may induce an imbalance of nutrient ratios, such as N:P ratio (Krüger et al., 2020; Sardans et al., 2016). However, the observational data on atmospheric P deposition are still limited for forest areas (e.g., Chiwa, 2020; Du et al., 2016) and N‐P imbalances have been reported from various regions (Boccuzzi et al., 2021; Krüger et al., 2020; Peñuelas et al., 2013). Taking into account the global pattern of N and P limitation in forest areas (Du et al., 2020), N and P deposition should be monitored together. Both N and P cycles are listed as important Earth‐system processes in the concept of “Planetary boundaries” with N cycle already transgressing its boundary (Rockström et al., 2009; Steffen et al., 2015).

Climate has an important role in regulating the global patterns of terrestrial N and P limitation (Du et al., 2020). Specifically, there is a shift from relative P to N limitation at lower mean annual temperature, temperature seasonality, mean annual precipitation, and higher precipitation. Future climate change will likely reshape the spatial pattern of nutrient limitation. For instance, climate warming will improve N availability at mid‐to‐high latitudes via increasing biological N fixation and N mineralization (Zaehle et al., 2010). Moreover, growth stimulation by rising atmospheric CO_2_ concentration ([CO_2_]) will increase nutrient demand and, in turn, result in greater nutrient limitation (Collalti et al., 2018; Wieder et al., 2015). The changing nutrient status under climate change will likely interact with the effects of S and N deposition and thus they should be considered simultaneously when projecting future forest dynamics.

## 4 GROUND‐LEVEL OZONE IMPACTS

Background O_3_ concentrations have increased throughout the last century due to the rising anthropogenic emissions of O_3_ precursors from fossil fuel and biomass burning (Cooper et al., 2014; Monks et al., 2015), although volatile organic compounds (VOCs) also are major precursors (Wei et al., 2014). Despite the decreasing trend of other air pollutants in the last decades (e.g., S and N compounds, heavy metals), global‐scale background O_3_ concentrations increased (Jakovljević et al., 2021; Sicard, 2021), but slight regional‐scale decreases in peak concentrations were observed (Schaub et al., 2018). Thus, O_3_ is nowadays one of the main phytotoxic air pollutants with the potential to affect forest ecosystems worldwide (Agathokleous et al., 2020; Bytnerowicz et al., 2016; De Marco et al., 2020; Feng, Shang, Gao, et al., 2019; Sicard, Augustaitis, et al., 2016).

**FIGURE 3:**
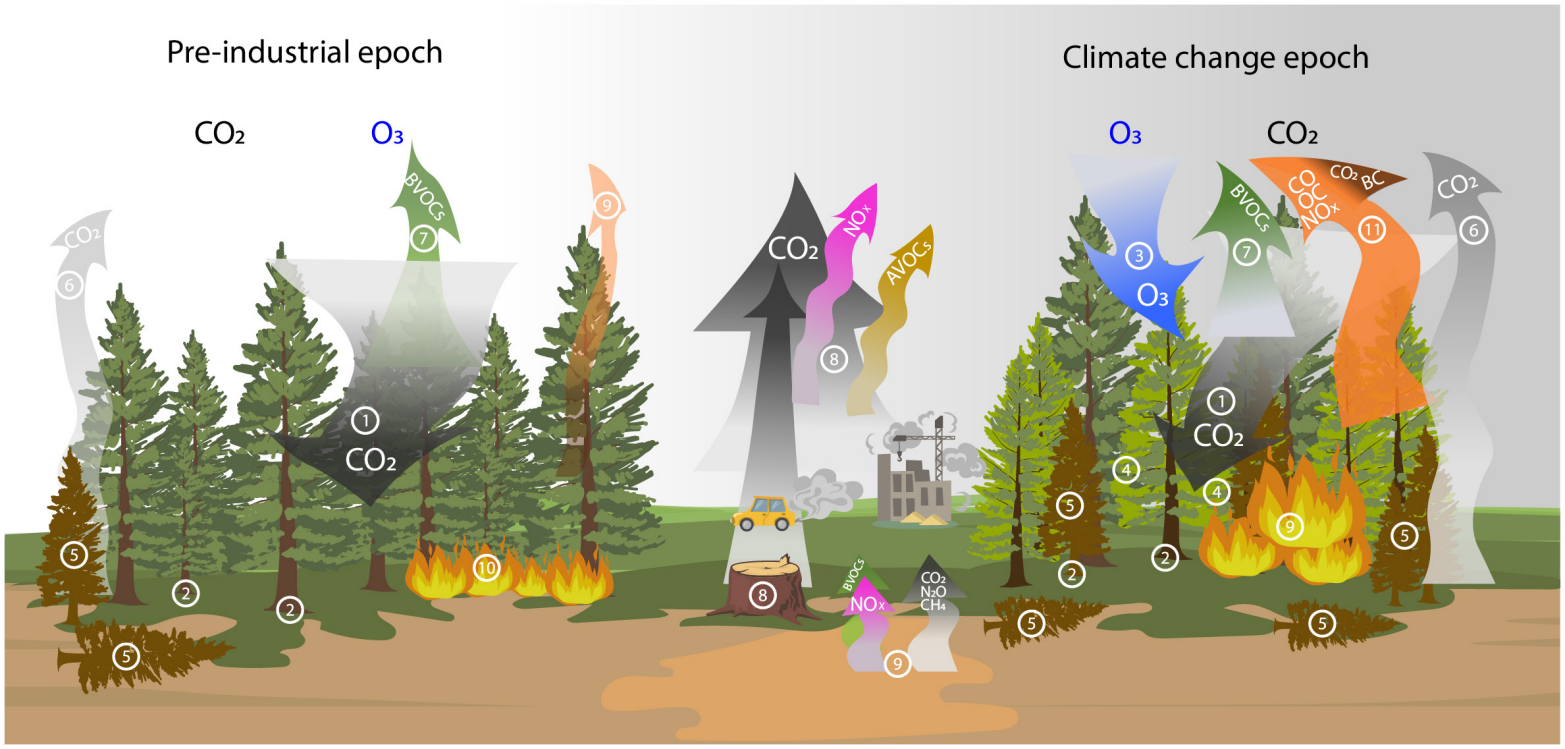
The Gordian Knot of the Forest–Ozone–Carbon interactions. In the pre‐industrial epoch, carbon is stored via photosynthesis (1) and leads to long‐term carbon sequestration into aboveground and belowground (roots and soil) wood biomass (2) (Agathokleous et al., 2016; Grantz et al., 2006). The higher CO_2_ levels, alone, in the atmosphere are expected to “feed” forest growth (Koike et al., 2018) and have beneficial effects. The increased O_3_ levels, alone, depress forest trees, contributing to “forest decline syndrome,” that is, visible injury, photosynthesis, carbon sequestration, carbon storage changes (7), and biomass decay, which also releases CO_2_ in the atmosphere (8) (Agathokleous et al., 2016; Sandermann et al., 1997; Sicard et al., 2021; Takahashi et al., 2020). In a positive feedback, the depressed forest vegetation emits more BVOCs (4), further increasing O_3_ levels (Peñuelas & Staudt, 2010). Concurrent elevated concentrations of CO_2_ end O_3_ may outcome to a sustained increase in Net Primary Productivity (NPP), while the adverse long‐term effect of increased O_3_ on NPP may be lesser than projected (Talhelm et al., 2014). Elevated CO_2_ levels negate or even overcompensate the negative O_3_ effect on ecosystem functions and the cycles of carbon and nitrogen. Anthropogenic emissions of CO_2_, NOx, and volatile organic compounds (VOCs) (3) as well as biogenic VOCs (BVOCs) emitted by forests (4) contribute to increased O_3_ levels in the atmosphere (Yu & Blande, 2021). Soil microbial processes contribute to soil‐emitted BVOCs and NOx (O_3_ precursors; Gray et al., 2010) as well as CO_2_, N_2_O and CH_4_ (Yao et al., 2009; Zhang et al., 2021) (5). Under advanced climate change, forest fires are expected to be more frequent and larger than in the pre‐industrial epoch (Zhang et al., 2021). These fires release carbon monoxide (CO), organic carbon (OC), NOx (all of which contribute to O_3_ formation), and black carbon (BC; which influences photosynthesis by increasing diffuse radiation) as well as CO_2_ (which further intensifies global warming; Flannigan et al., 2009; Kumar et al., 2019; Pellegrini et al., 2021; Yue & Unger, 2018) (9).

Ozone burdens are higher in the Northern (O_3_ mean concentration 35–50 ppb) than in the SH (O_3_ mean concentration <20 ppb; Sicard et al., 2017). For example, widespread O_3_‐induced visible injury, a specific damage associated with O_3_ exposure, was found at 17 forest plots in Europe (Paoletti et al., 2019; Sicard et al., 2020). The NH is more covered by land and terrestrial ecosystems, and more inhabited by humans than the SH, and thus is more affected by anthropogenic activities. However, the SH is less monitored and thus O_3_ burdens and effects may be underestimated. While there are hundreds of papers on O_3_ effects on forest plants and forests in the NH (i.e., Agathokleous et al., 2015; Feng, Shang, Gao, et al., 2019; Izuta, 2017; Sicard et al., 2020) indicating various effects of O_3_ in interaction with climate change (Figure 3), relevant research in the SH remains scarce.

The analysis of O_3_ effects in Latin America is complex due to the enormous diversity of natural and agricultural ecosystems. Most monitoring studies on O_3_ effects on forest plants conducted in the SH come from Brazil. Urban and industrial development has been more intense along the Atlantic Brazilian coast, especially in Southeastern region. Consequently, more severe O_3_ effects on the Atlantic forest located in this subtropical region (mainly São Paulo and Rio de Janeiro States) are expected (Domingos et al., 2003; Moura et al., 2014, 2018). Ozone effects on native tree species from the Atlantic Forest have recently been determined in the field or experimentally, pointing to distinct tolerance levels and highlighting the need to expand knowledge on this topic (Cassimiro et al., 2016; Engela et al., 2021; Fernandes et al., 2019; Moura et al., 2018). In the SH, the Amazon spans over 629 million hectares of rainforest, accounting for 54% of the total rainforests left on Earth (Peng et al., 2020). Recent modeling approaches have shown O_3_ concentrations have increased above the Amazon and Cerrado biomes in Brazil as a response to biomass burning and regional air pollution (Gerken et al., 2016; Pope et al., 2019). The lowest O3 exposures reported are in Australia, New Zealand, southern parts of South America, and some northern parts of Europe, Canada, and the United States (Mills et al., 2018; Sicard et al., 2017). However, unfortunately, a proper O_3_ monitoring network does not currently exist. Despite the presence of ground‐level O_3_ monitoring networks in all the developed countries (Lefohn et al., 2018), there is still a lack of an integral network of ground‐level O_3_ monitoring across Asia, although 1500 monitoring stations have recently been installed in China (Feng, Shang, Gao, et al., 2019).

Another challenge in monitoring O_3_ impacts on forests is the choice of metrics. The AOT40 index (Accumulated Ozone over Threshold of 40 ppb ozone), describing the exposure of plants to high O_3_ concentrations, is the default measure for policy directives of the European Union (Directive 2008/50/EC). However, AOT40 has been criticized because it is not a proxy of gas uptake through leaf stomata (stomatal flux), and flux‐based indices have been applied (Anav et al., 2022; De Marco & Sicard, 2019; Paoletti et al., 2019; Sicard et al., 2020) and showed O_3_ risks to vegetation would be different from AOT40 (Anav et al., 2016; De Marco et al., 2015). The new standard developed in Europe (Emberson et al., 2000) is the stomatal O_3_ flux, defined as POD (Phytotoxic Ozone Dose). This standard depends not only on O_3_ concentration, but also environmental (e.g., light intensity, air temperature, relative humidity, soil moisture) and plant conditions (phenology, leaf morphological, and physiological traits). A major impact of O_3_ is reduced aboveground and belowground carbon sequestration of forests (Agathokleous et al., 2016; Gao et al., 2017; Figure 2). Ozone effects on biogenic volatile organic compounds (BVOCs) are complex, as some compounds may decrease (e.g., isoprene) while other compounds increase (e.g., monoterpenes; Feng, Yuan, et al., 2019). Different BVOC compounds have different capacity to generate O_3_, with isoprene having higher O_3_‐forming potential than monoterpenes (9.1 g 0_3_ (g VOC)^−1^ and 3.8 g 0_3_ (g VOC)^−1^, respectively; Benjamin & Winer, 1998). However, sesquiterpenes and some monoterpenes also contribute to the removal of O_3_ at the canopy level and play an important role in the feedback between stress‐induced VOC emissions and O_3_ or aerosol formation (Calfapietra et al., 2013). The emission of isoprene, the most abundant BVOC, can also be decreased by drought and CO_2_ and increased by warming (Feng, Shang, Li, et al., 2019), indicating complex O_3_‐climate interactions that remain elusive in real‐world forests. Soil microbial processes contribute to emission of BVOCs and NO_x_ that act as O_3_ precursors (Gray et al., 2010). Overall, soils play an important role in forest VOC exchange, defining also carbon storage by forest ecosystems, and fluxes depend upon BVOC compounds and vegetation types (Mäki et al., 2019; Rinnan & Albers, 2020; for details and values of fluxes in different vegetation types and environmental media, see also Tani and Mochizuki (2021)). However, the specific contribution of soil in VOC exchanges and O_3_ formation remains poorly understood.

## 5 TRACE ELEMENTS AND RADIOACTIVE CONTAMINATION OF FOREST ECOSYSTEMS

Heavy metal pollution was an important subject in widespread forest decline in the 1980s–1990s (Gawel et al., 1996), but more recently has become a major item in phytoremediation (Pulford & Watson, 2003) and environmental monitoring (Godzik, 2020). The term “heavy metals” is now discouraged, and these elements are now included more broadly as “trace elements” (Pourret & Bollinger, 2018). Trace elements are a major component of particulate pollution (Antoniadis et al., 2017; Grantz et al., 2003; Li et al., 2015; Schlutow et al., 2021; Tóth et al., 2016). At the global scale, trees are important for their role in retaining particulates (Yue et al., 2021). Nevertheless, in some regions, soil contamination by trace elements remains so high that it continues to kill trees and prevents natural recovery (Kozlov et al., 2009). Among trace elements, radionuclides display the most phytotoxic potential.

The use of nuclear energy or nuclear applications in health, agriculture, environmental management, or industry/military resulted in releases of radionuclides into the environment (Hong et al., 2012). The first large‐scale radioactive contamination from anthropogenic sources occurred through global radioactive fallout from nuclear weapons' tests conducted in the atmosphere during 1945–1980 (Aoyama et al., 2006; United Nations, 2000). A variety of long‐ and short‐lived radionuclides were released during nuclear incidents; in particular ^137^Cs with a relatively long half‐life (~30 years) compared to other radionuclides, such as ^134^Cs and ^131^Cs. Other major releases of radionuclides occurred from the Chernobyl nuclear power plant accident in 1986 (International Atomic Energy Agency, 2006) and from the Fukushima Daiichi Nuclear Power plant accident in 2011 (Chino et al., 2011; Terada et al., 2020; Yoshida & Takahashi, 2012).

**FIGURE 4:**
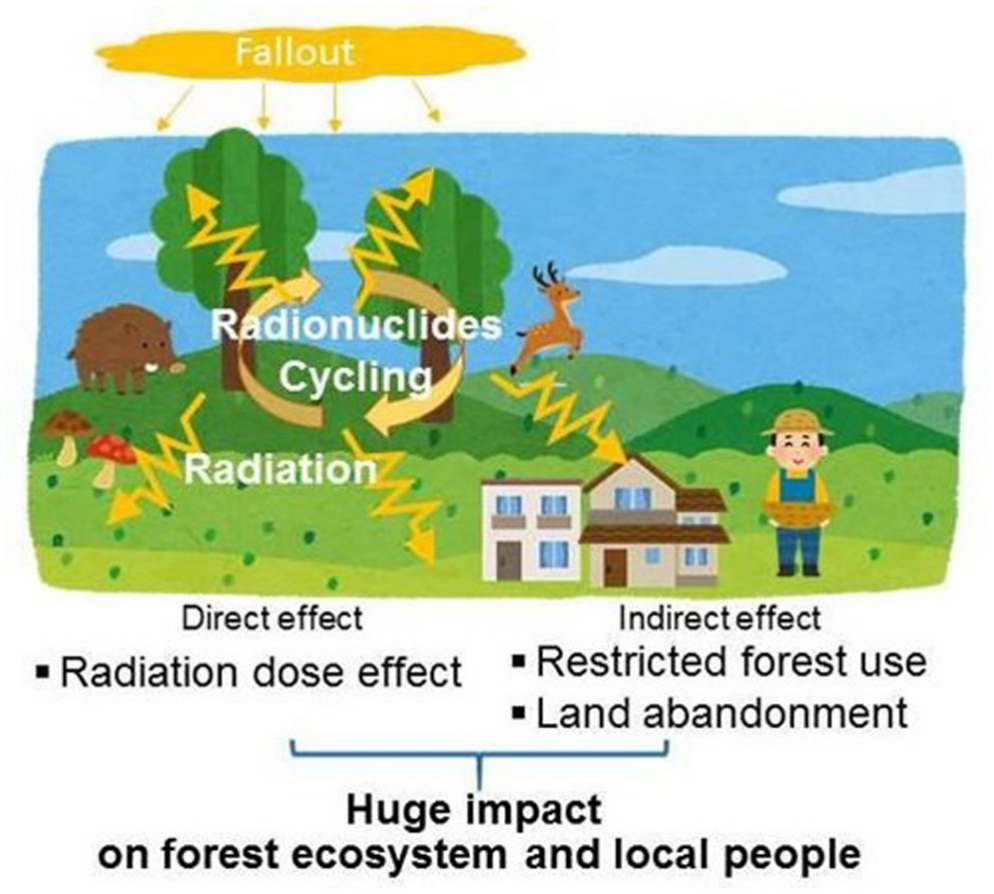
Diagram of direct and indirect effects of forest radioactive contamination. The deposited radionuclides remain in the forest and continue to circulate in the forest ecosystem, and radiation can have adverse effects on forest biota (direct effects). Restrictions on forest use and land abandonment to avoid exposure can also affect forest ecosystems, including changes in vegetation and wildlife populations. It has direct and indirect impacts on ecosystems and local residents.

Radioactive contamination of forests has different types of impacts (Figure 4). First, direct radiation can affect trees and animals and occur at the level of DNA, cells, individuals, population to whole ecosystems, and ranges from reparable DNA damage to death of organisms (Committee on the Biological Effects of Ionizing Radiation, 1990). An example of direct impacts of high radiation doses to trees is the “Red forest” in the Chernobyl exclusion zone, where pine trees became reddish brown and died following the accident (Beresford et al., 2016). Another visible impact of radiation exposure in trees is the occurrence of morphological abnormalities (Watanabe et al., 2015; Yoschenko et al., 2011, 2016). Compared to the effects caused by high doses of radiation, those potentially caused by relatively lower radiation dose are confounded by many other factors and are still not clearly understood (Beresford et al., 2020; Ji et al., 2019; Strand et al., 2017). In exposed areas, forest ecosystems are released from pressure by human existence, resulting in creation of ecological niches and expansion of populations of some species (Deryabina et al., 2015; Lyons et al., 2020; Perino et al., 2019). Through intensive monitoring, it was confirmed that the overall dynamics of ^137^Cs within forest ecosystems were similar between Chernobyl and Fukushima: tree canopies captured the deposition of ^137^Cs and ^137^Cs migrated from the canopy to the soil surface via water and litter fall, and most of it stays in the top layers of soil (Itoh et al., 2015; Kato et al., 2019; Suchara et al., 2016). However, the migration velocity and distribution patterns of ^137^Cs within forests and tree bodies differ substantially among forests and trees (Imamura et al., 2017; Ohashi et al., 2017). It is essential to continue experimental studies to identify the key processes influencing ^137^Cs dynamics in forest systems, such as soil potassium concentrations and fixation processes within soils (Kobayashi et al., 2019; Manaka et al., 2019). Various models have been developed to characterize ^137^Cs dynamics in forests; however, improvements are necessary to reproduce variations between forest types and species compositions (Hashimoto et al., 2020). Another aspect of radionuclide pollution is that deposited radionuclides, which are easy to detect and measure, provide an unintentional but useful opportunity to track biogeochemical cycles in forest ecosystems (Fukuyama et al., 2008).

## 6 COMBINED EFFECTS OF MULTIPLE FACTORS ON FOREST ECOSYSTEMS

Our knowledge on combined effects of multiple factors on ecosystem health originated primarily from temperate and boreal forests of North America and Europe and is limited for tropical forests, especially of those in Africa (Matyssek et al., 2017). In other words, areas that have recently experienced the highest risk of forest degradation are studied to a lesser extent than the areas where risk is low. In addition, many communities whose food security and wealth generation critically depend on forests are located in geographic regions where our understanding of factors affecting forest ecosystem health is poor. This geographic bias is typical for ecological and environmental research (Archer et al., 2014), and its consequences are generally seen as severe, because results obtained with one study system may appear of little use in predicting the responses of another, geographically distinct, study system (Haukioja et al., 1994).

Air pollution levels may become more harmful for plants as the climate warms (Zvereva et al., 2008, 2010). More multi‐factorial manipulative studies are needed because effects of two or more co‐occurring factors on tree growth and forest productivity cannot be adequately predicted from single‐factor experiments (Niinemets, 2010). The combined effects of two major abiotic aspects of global change, mostly changes in CO_2_ and warming, on growth of forests are studied in detail (Baig et al., 2015; Curtis & Wang, 1998; Zvereva & Kozlov, 2006), and suggest air temperature may modify plant responses to elevated CO_2_. Across 42 experiments with woody plants, aboveground biomass increased significantly with both CO_2_ (the so called “fertilization effect”) and air temperature (by 21.4% and 18.1%, respectively), whereas these two factors acting simultaneously showed a much smaller effect (8.2%) because of compensating effects (Baig et al., 2015). Nitrogen fertilization enhances the biomass response to elevated CO_2_ (Parrent & Vilgalys, 2007) despite not universally (Terrer et al., 2019). The type of mycorrhiza was also an important factor related to the effects of soil nutrient availability on elevated CO_2_‐induced growth enhancement (Baig et al., 2015). However, two‐factorial experiments involving both O_3_ exposure and elevated CO_2_ are limited. Several studies under elevated CO_2_ showed a reduction in the negative effects of O_3_ because elevated CO_2_ induced stomatal closure leading to lower O_3_ uptake (Grams et al., 1999; Watanabe et al., 2017). In contrast, the addition of N alone exacerbated negative effects of O_3_ on photosynthesis of trees (Feng, Shang, Li, et al., 2019), while exposure to drought stress did (Gao et al., 2017) or did not protect plants from O_3_‐induced effects (Alonso et al., 2003, 2014).

Forest health also can be compromised by insect herbivory, including both devastating outbreaks of forest pests and changes in background herbivory. Despite relatively low levels of plant damage (5%–7% of leaf biomass annually: Kozlov et al., 2015), background herbivory greatly reduces growth of woody plants (Shestakov et al., 2020; Zvereva et al., 2012). Although warming, drought, CO_2_ increases, N deposition, and air pollution were repeatedly found to increase herbivory (Lincoln et al., 1993; Logan et al., 2003), these conclusions were likely affected by research and publication biases (Zvereva & Kozlov, 2010) and/or were derived from results of short‐term laboratory experiments, which tend to overestimate the effects relative to natural ecosystems (Bebber, 2021). Within forest ecosystems across the globe, no increase in insect herbivory was observed from 1952 to 2013 (Kozlov & Zvereva, 2015). Similarly, long‐term monitoring did not reveal the effects of either pollution‐induced disturbance or 2.5°C climate warming on insect herbivory in subarctic birch forests (Kozlov et al., 2017). Thus, the evidence regarding combined effects of climate warming and air pollution on insect herbivory remains somehow contradictory.

Other factors whose effects on forest trees have been studied in multi‐factorial studies include (but are not limited to) cattle/deer grazing, harvest of non‐timber forest products, drought, flooding, soil salinization, spring frost, heat waves, and increased ultra‐violet radiation (e.g., Mac Nally et al., 2011; Pliūra et al., 2019; Sugai et al., 2019; Varghese et al., 2015). However, a low number of such studies precludes any generalization regarding effects of these factors, combined with CO_2_ and air temperature increases or O_3_ and insect herbivory on health of forest ecosystems. Modeling studies jointly assessing the effects of climate change and air pollution can greatly help for understanding and predicting future developments of forests (Akselsson et al., 2016; Dirnböck et al., 2017; Etzold et al., 2020; Fleck et al., 2017; Rizzetto et al., 2016).

## 7 GENETIC INFORMATION RELATED TO PHENOTYPES AND PHYSIOLOGY OF FOREST TREES

Air pollution, climate change, increased pests and pathogens, land‐use changes, and forest fragmentation can all reduce genetic diversity and make forests more fragile and sensitive to other threats (Gauthier et al., 2015). Current vegetation and forest growth models are largely parameterized on direct growth and gas exchange measurements or remote sensing, while information from biological and genetic regulation mechanisms are still scarce. For example, part of the carbon fixation products (i.e., photosynthates) that is not used for biomass production is released in soil as root exudates, some is stored, and some organic carbon is emitted as BVOCs affecting plant and community ecology and atmospheric chemistry (Blande, 2021; Collalti et al., 2020; Maja et al., 2015; Naidoo et al., 2019; Šimpraga et al., 2019). Carbon sink strength of trees is known to be impaired by limitations in water and nutrient availability, heath spells, air pollutants, and increased herbivory. However, plant defense processes against different abiotic and biotic factors are complex and involve multiple signaling pathways (He et al., 2018), potentially affecting how carbon is allocated to different organs (Merganičová et al., 2019). Most of the underlying resistance mechanisms are described or predicted from short‐living herbaceous model systems, whereas investigations on mechanisms of defense and adaptation of forest trees are much more challenging due to long lifetime, high genetic diversity, and variation of growth environments and climates (Naidoo et al., 2019). There is an urgent need to intensify studies on the mechanisms underlying the resilience of forest ecosystems to current and long‐term effects of air pollution and climate change, utilizing genetic, species, and ecosystem‐level functional diversity as well as adaptive management, resistance breeding, and genetic engineering (Naidoo et al., 2019). Mechanistic understanding is increasingly important also for efforts in afforestation and protection of primary forests. In principle, there are two main approaches for achieving resistance in forest trees: (i) selection of resistant phenotypes identified in field experiments (Sniezko & Koch, 2017) or polluted sites (Eränen et al., 2009; Kozlov, 2005); and (ii) structured breeding programs relying on multitude of omic techniques (Naidoo et al., 2019). The databases for genetic information of tree species have been rapidly increasing, and the most important model systems for forest trees are *Populus*, *Eucalyptus*, *Quercus*, *Castanea*, *Pseudotsuga*, *Pinus*, *Picea*, and *Betula* genuses (Falk et al., 2018; Salojärvi et al., 2017). Genetic engineering efforts by forest biotechnology companies have produced transgenic *Eucalyptus* and *Populus* trees with enhanced growth and disease‐resistant properties (Naidoo et al., 2019). Silver birch (*Betula pendula* Roth) is an excellent model system for elucidating the adaptation and acclimation capacity of forest trees to rapidly changing climate due to its (i) wide latitudinal and longitudinal distribution; (ii) recent advances in population genomics and evolutionary history of birch species (Salojärvi et al., 2017); and (iii) existence of well‐characterized birch genotypes that have been intensively studied for C and N economy, photosynthetic efficiency, metabolism, chemistry, and phenology (Deepak et al., 2018; Tenkanen et al., 2020). The population genomic analyses of silver birch provide insights on natural selection mechanisms, with candidate genes relevant for adaptation of trees to changing environment, biotic stress, and growth regulation (Salojärvi et al., 2017). Studies with birch have also shown the C‐sink strength of trees cannot be explained by physiological or genetic approaches alone, but there are many negative and positive interactions with pollutants, climate, pests, pathogens, microbiomes, and between plants that should be understood in more detail (Naidoo et al., 2019; Silfver et al., 2020; Wenig et al., 2019).

Plant phenotypes are strongly affected by the environment, and often genotype *per* environment interaction is the factor of greatest interest. Methodologies have been developed for non‐destructive forest‐level and individual tree‐level phenotyping with remote sensing techniques, which are particularly useful for identifying superior genotypes under different stress conditions (Dungey et al., 2018; Kefauver et al., 2012; Ludovisi et al., 2017). Recent advances in metagenomics and the increasing knowledge of the importance of microbiomes in plant health offer new opportunities for forest health management (Imperato et al., 2019; Naidoo et al., 2019; Wenig et al., 2019). The regulatory networks of forest trees and the beneficial non‐pathogenic microbes living around and on the surfaces of plant roots (rhizosphere), leaves (phyllosphere), or in the internal plant tissues (endosphere) can be particularly important for carbon and nutrient dynamics of trees and the development of tree immunity (Naidoo et al., 2019). Microbes are known to help plants in water and nutrition acquisition, defense against pathogenic microbes, tolerance to abiotic stress, adaptation, promotion of the establishment of mycorrhizal association, and plant growth regulation, forming a holobiont system with host trees (Imperato et al., 2019; Naidoo et al., 2019; Wenig et al., 2019). Fungal and bacterial communities in forest soils have been shown to respond to changes in climate with a shift in their community composition as well as in their diversity (Dubey et al., 2019; Jansson & Hofmockel, 2020; Milović et al., 2021; Simard, 2010). For example, under elevated CO_2_, we can observe alteration in relative abundances of bacteria and increased bacterial to fungal ratio (Dubey et al., 2019), as well as an increase in ectomycorrhizal colonization rate but a decrease in ectomycorrhizal diversity (Wang et al., 2015). Warming and elevated O_3_ reduced ecto‐ and arbuscular mycorrhizal colonization and shifted arbuscular mycorrhizal community composition in favor of the genus *Paraglomus*, which has high nutrient‐absorbing hyphal surface (Qiu et al., 2021; Wang et al., 2015). At the same time, exposure to higher levels of O_3_ is associated with lower soil microbial biomass and with changes in the overall structure and composition of poplar rhizosphere soil microbial communities (Li et al., 2021). The decreased growth of roots and decrease in ectomycorrhizal colonization rate and a shift in species abundance might be an early indicator of the damaging impacts of O_3_ in some tree species, occurring prior to visible responses of aboveground tree parts (Katanić et al., 2014).

## 8 MODELING FOREST ECOSYSTEMS FOR RISK ASSESSMENT

Scientific methods in forestry, including empirical models of tree growth, were primarily used for optimization of timber harvest throughout the 20th century (Porté & Bartelink, 2002). A more integrative modeling approach, acknowledging natural disturbances (e.g., wind, fires, pests, diseases) as inherent elements of forest ecosystem dynamics, was developed when computational advancements allowed for the integration of greater complexity (Blanco et al., 2020; Perera et al., 2015), although some biotic factors of forest disturbance such as herbivory are still rarely modeled (De Jager et al., 2017). Since the forest dieback and acidification debate in Europe in the 1980s, large efforts were put into improving understanding and prediction of anthropogenic disturbances on biogeochemical dynamics of forests. Starting from models mainly targeting the fate and effects of acid rain in forest ecosystems (Nilsson, 1988; Sverdrup & De Vries, 1994), simulation tools have broadened to include other pressures, such as N deposition (De Vries et al., 2010), O_3_ (Hoshika et al., 2015), and climate change and forest management (Collalti et al., 2018). Modeling has been demonstrated to be a valuable tool for studying forest responses to present and future disturbances, allowing ecologists and foresters to deal with the study of complex interactions and to evaluate future management strategies (e.g., Collalti et al., 2018; Fleck et al., 2017) or policy options (e.g., Belyazid et al., 2010; Dirnböck et al., 2018).

Existing relationships between forest structure and composition and environmental variables were initially used to build empirical models that describe past ecosystem behavior and extrapolate to future conditions (Gustafson, 2013). Subsequent modeling efforts simulated the causal biogeochemical mechanisms that underlie the responses of ecosystems to these environments (Kimmins et al., 2008). These so‐called process‐based models (PBMs) study the ecological processes and are considered one of the most reliable approaches for modeling forest ecosystem dynamics under global change (Evans, 2012; Maréchaux et al., 2020). However, forecasting forest growth is still a priority in many studies, either for planning forestry activities under air quality and climate change scenarios or as part of carbon storage calculations (Blanco et al., 2020).

**FIGURE 5:**
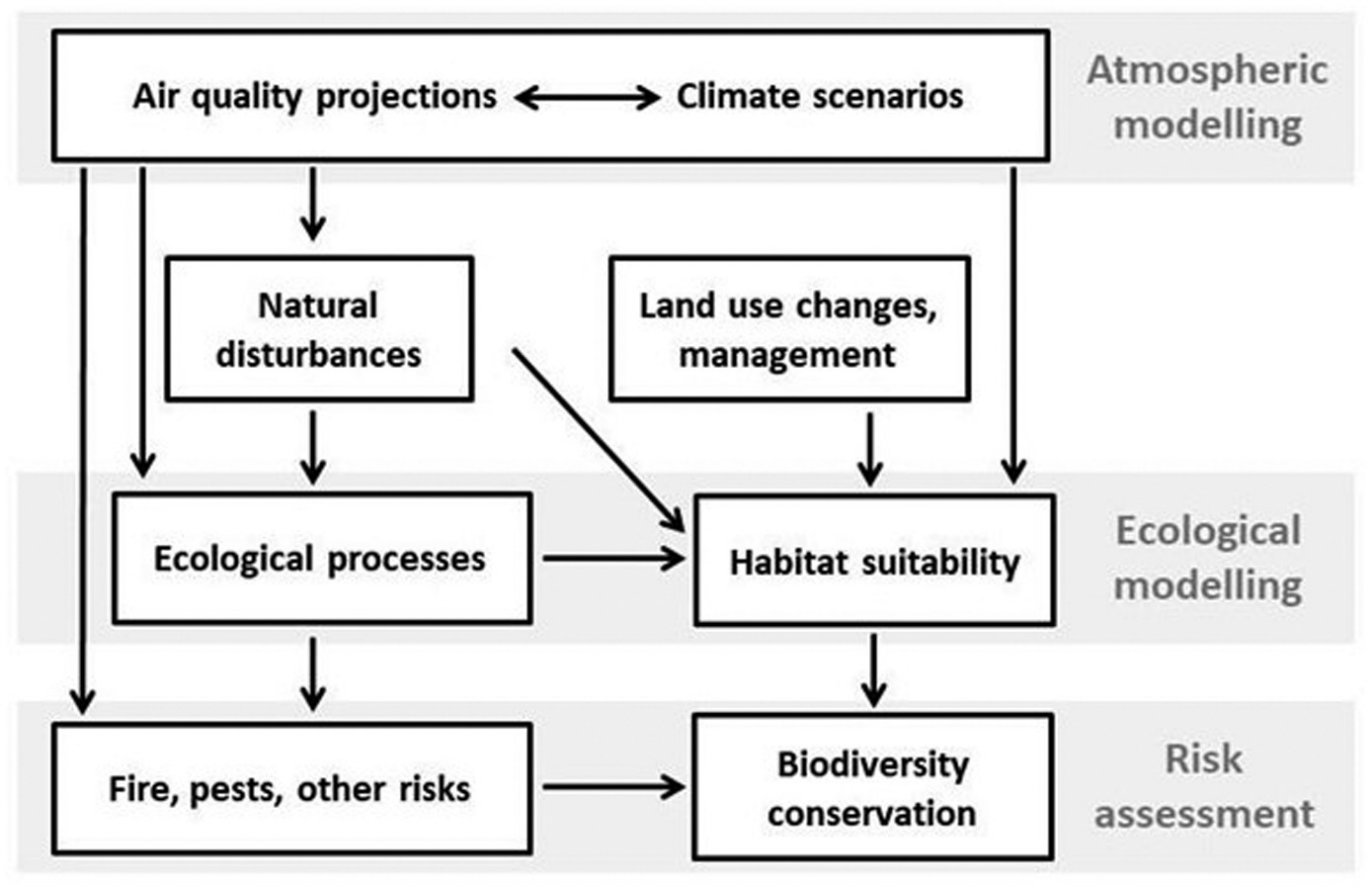
Simplified flux of information diagram in modeling approach for risk assessment of air pollution and climate change. The modeling and risk assessment process might be as complex as the modelers need and the availability and suitability of models and data allow. There are three main blocks (grey bands): atmospheric and ecological models (top two) are the tools to reach the objective of risk assessment (bottom). Models are used both in the internal processing and description of the information contained in the boxes, and in the transmission of information between them (model inputs and outputs). For example, habitat suitability can be modeled using vegetation response models (such as VEG; Belyazid et al., 2019 or PROPS; Dirnböck et al., 2018), that are particularly designed to process output from process‐based models as input information, but it can be also modelled by species distribution models that are particularly designed for large‐scaled input datasets (e.g., Noce et al., 2017). Information generally needed in any environmental study (such as soil and terrain variables) has been obviated in this diagram.

The general trend toward biodiversity conservation in international policies (e.g., EU Biodiversity Strategy for 2030; European Commission, 2020), its importance in preserving ecosystem services, and the use of biodiversity metrics as indicators in risk assessment (Coordination Centre for Effects, 2017) and policy evaluation (Hein et al., 2018) make the simulation of species composition changes a decisive function for any model. When dynamic PBMs are used for forecasting biodiversity shifts, they are usually combined with vegetation response models based on species niche suitability and competition (Belyazid et al., 2019; Dirnböck et al., 2018). PBMs have been used at stand (e.g., Collalti et al., 2016), landscape (Shifley et al., 2017), regional (Belyazid et al., 2019; De Marco et al., 2020; Santini et al., 2014), and global scales (e.g., Krause et al., 2017). However, their implementation is restricted at larger scales since PBMs need large, detailed input datasets, which are often not available at national or continental scales. At these scales, a currently suitable approach is using new models, mostly empirical, based on currently available large datasets, such as species distribution models (SDMs; Maréchaux et al., 2020; Noce et al., 2017). In the same way that PBMs rose with the increasing computational power during the last decades of the 20th century, SDMs have improved during the present decade, in parallel to the increase in web available, reliable spatial‐referenced data, including environmental and meteorological data, forest inventories, habitat distribution, aerial images, and remote sensing (Pecchi et al., 2019; Urban, 2015). SDMs are usually statistical models that are currently used to support sustainable planning of forests at national and international scales (Zang et al., 2012), with correlative SDMs using maximum entropy algorithms being most frequently used (Noce et al., 2017; Pecchi et al., 2019). Using forest decision support systems, climate change scenarios and the balance of delivered ecosystem services can be suggested as a methodological framework for validating forest management alternatives aiming for more adaptiveness in sustainable forestry (Marano et al., 2019; Mozgeris et al., 2019). Moreover, some of the vegetation models associated with PBMs to assess or forecast biodiversity are SDMs that may be applied from site to regional scales (e.g., Wamelink et al., 2020). There are some recent examples of SDMs implemented to assess forest biodiversity response to atmospheric pollution and climate change, such as Hellegers et al. (2020) and Wamelink et al. (2020). However, these models still lack essential information to feed their predictions, since new field observations and experiments with novel set‐ups (e.g., Hansen & Turner, 2019) are needed to address the potential successional and disturbance dynamics under the forthcoming climate conditions (McDermott, 2020). Therefore, there are several possible approaches for different problems that scientists and managers must deal with (Blanco et al., 2020; Fabrika et al., 2019; Maréchaux et al., 2020). The modeling process might be as complex as needed by risk assessment objectives (Figure 5), providing models and data are available and suitable. In general terms, empirical models are good at predicting biomass and forest structure in the shorter term, and consequently producing good management recommendations for the present conditions, but are not reliable in novel situations (i.e., future air pollution and climate change). PBMs are good at studying effects and underlying processes of change, particularly in the context of global change. However, they still have low feasibility at broad scales, and the calibration and validation processes are highly time‐consuming (particularly for the less‐modeled species or regions). SDMs are appropriate for early risk assessment on biodiversity conservation at the broadest scales, but still too empirical, which diminishes their reliability in the long term (Urban et al., 2016). Mixing process‐based with empirical approaches (hybrid models), integrating, and connecting different models (meta‐ and mega‐models; Blanco, 2013) are excellent strategies to answer specific questions.

## 9 NEW DIRECTIONS: INTEGRATING EXPERTS' OPINIONS

### 9.1 Air pollution monitoring network

While conventional field‐based monitoring plots will continue to dominate the mainstay of air pollution and climate change research in forests, they are costly and often logistically difficult to conduct over large areas. Therefore, remote sensing techniques will be more and more appropriate for large‐scale monitoring programs, even though a more in‐depth approach still needs to be developed. Finer temporal intervals are required for in‐depth understanding of some responses (e.g., stomatal O_3_ fluxes require a continuous‐monitoring approach). Highly instrumented field sites are now cheaper and technically affordable. Integrating data from transcontinental long‐term ecological research infrastructures in tree‐based models would lead to a better understanding of how ecosystems work (Fischer et al., 2011). Long‐term data series can be integrated in existing big databases such as the Global Atmosphere Watch (GAW) Program and the international Tropospheric O_3_ Assessment Report (TOAR; Schultz et al., 2017; WMO, GAW, 2003). These raw databases can lead to the development of new products for temporal and spatial analysis (data analysis, maps of data distributions, and data summaries) that are freely accessible to the scientific community and other stakeholders. Such databases can be used as tools for mechanistic and diagnostic understanding and upscaling.

The need for a global forest monitoring is irrefutable, and “supersites” promote the integration of research communities in a transcontinental collaboration network by upgrading existing ground‐based observation networks (e.g., FLUXNET, ICP, NEON) covering all biogeographic areas (e.g., tropics, subtropics) and ecosystem types (e.g., woody savannas).

### 9.2 Elements deposition in forests

The effects of S and N deposition on forest health have been reducing gradually in many regions but problems have not been solved. Legacy S pools remain, which could be affected by changing climate. Reduction of S deposition is associated with reduction of base cation deposition, which may alter nutrient status and increase the risk of further soil acidification. The total inorganic N deposition has been declining due to the implementation of air pollution control policies, but the relative importance of NH_3_ emissions and deposition is now higher (Butler et al., 2016; Du, 2016), showing a relative increase of 0.38% per year over the period 1985–1999 (Du, 2016). Since ecosystem responses to declining N deposition may show hysteresis (Gilliam et al., 2019) and key mechanisms of the N‐induced changes in forest ecosystems are not fully understood (Lilleskov et al., 2019), long‐term monitoring of N‐, S‐, and P‐cycles and base cations deposition should be studied together to better understand biogeochemical processes and plant biodiversity under climate change. Moreover, interactions between nutrient deposition and rising O_3_ concentrations should be considered in future studies (Shi et al., 2017). Long‐term monitoring should be continued even after significant air pollution reductions to capture and understand the potential long‐term effects of pollution and ecosystem recovery.

### 9.3 Ground‐level ozone

Surface O_3_ concentrations are generally higher in rural areas than in urban areas (Sicard, 2021). However, as O_3_ levels are rising in cities (Sicard, 2021), special attention should be paid to urban and peri‐urban forests, which offer services to local communities (Bruckman et al., 2016) and can help meet air quality standards in cities (Sicard et al., 2018). Because forest tree species play important (species‐dependent) dual roles as sinks and sources of O_3_ precursors (Geng et al., 2011; Saitanis, Agathokleous, et al., 2020), the O_3_ forming potential (OFP) of the best regionally adapted forest tree species should be investigated and taken into account by decision‐makers to select species with lower OFP for urban planning (Sicard et al., 2018).

The observed high O_3_ burdens, their high spatial heterogeneity, and the differential susceptibility of forest tree species to O_3_, as well as their dual role as O_3_ sinks and precursor sources (Agathokleous et al., 2020; Li et al., 2018), suggest an urgent need for the establishment of a globally denser O_3_ monitoring network in natural forest ecosystems in particular in the SH. A new approach to the global O_3_ monitoring network and alternative methods for monitoring O_3_ are feasible thanks to innovative technologies (Saitanis, Sicard, et al., 2020), which will help to understand combined effects of O_3_ with other emerging environmental factors. There is also an urgent need to generate flux‐based standards and critical levels for forest protection taking into account the sensitivity of dominant forest tree species. Because of its limitations, the AOT40 index should not be adopted as default for risk assessment (Agathokleous et al., 2019; Anav et al., 2022; Sicard, Augustaitis, et al., 2016). Finally, the development of countermeasures for controlling anthropogenic O_3_ precursor emissions is also urgently needed.

Further research is still needed to develop O_3_‐effect indicators related to other ecosystem services provided by forests such as biodiversity, soil protection, and water conservation. Nonlinear models should be used for establishing cause–effect relationships under experimental conditions (e.g., Agathokleous et al., 2019; De Marco et al., 2013).

### 9.4 Multiple stressors on forest ecosystems

For a better knowledge on combined effects of multiple factors on ecosystem health, the selection of tree species for future studies should account for their phylogenetic relatedness with already studied species. Ecological and environmental studies addressing the responses of tropical forests to combined effects of climate change and air pollution should be intensified, in particular in areas at higher risk of deforestation in the SH. This research domain is strongly biased toward temperate and boreal forests of the NH. The evolutionary changes in response to rising global CO_2_ levels and air temperature elevation are known to occur in some plants, but the contribution of evolutionary processes to the forest responses to steady CO_2_ and air temperature rises remains unexplored. Experimental studies, addressing combined effects of different abiotic factors on forests, should be intensified in the SH and should carefully select tree species to assure a better representation of taxonomic and functional diversity of the approximate 73,000 tree species now found on the Earth (Cazzolla Gatti et al., 2022).

### 9.5 Radioactive contamination of forest ecosystems

Despite many papers reporting radioactivity effects on forest ecosystems (Strand et al., 2017; Tamaoki, 2016), there is still no consensus on the mechanism through which radiation impacts forest ecosystems or the dose rates at which impacts begin to occur (Beresford et al., 2020; Strand et al., 2017). More robust and synthesis studies are essential to inform (i) key processes regulating the dynamics of radionuclides within forests; (ii) models for tracking radionuclides and prediction; (iii) holistic assessment of impacts caused by radioactive contamination and its countermeasure development; and (iv) use of ^137^Cs as a tracer. Furthermore, cost efficient forest countermeasures must be developed and decisions must include locals, scientists, stakeholders, and governments.

### 9.6 Genetic information of forest trees

More effort should focus on phenomics, combining high‐throughput capture of tree phenotypes, genotype information, data science, and engineering (Falk et al., 2018; Naidoo et al., 2019). Future work should include metadata integration and improved visualization for comparative genomics. Characterizing the root traits and phenotypes with association to genomics and shoot phenotyping is necessary for whole‐plant resistance breeding (Chuberre et al., 2018; Tracy et al., 2020; Wiley et al., 2020). Rhizosphere phenotyping opens new opportunities for experimental approaches, including stress treatments, repeatability and combined use of imaging techniques and machine learning to extract new traits from images, within a systems approach (Tracy et al., 2020). The belowground net primary production accounts for 40%–70% of total terrestrial productivity (Gherardi et al., 2020); therefore, more studies are needed to explore responses of tree roots to climate and pollution and quantify root losses to belowground herbivores.

### 9.7 Modeling and risk assessment

Model diversity constitutes a multi‐purpose toolkit that can help society to face the future challenges. Improving and enhancing scientific communication in forest modeling is required as part of this enterprise. The development of models integrating air pollution and climate change data from long‐term monitoring programs are needed to improve forest research assessing interactions between air pollution and climate change from the individual level to the stand level. Future challenges include understanding of (i) the impacts of air pollution on soil chemistry, (ii) the effects of climate change and air pollution on plant phenology and reproductive fitness, (iii) the capacity of forests to sequester carbon under changing, and extremes, climatic conditions and co‐exposure to elevated levels of pollution, and (iv) the effects of plant competitiveness (monocultures vs. mixed cultures, single trees vs. community responses) on plant responses to stressors.

## CONFLICT OF INTEREST

The authors declare no conflict of interest.

## Supporting information


Appendix S1.

## Data Availability

Data sharing is not applicable to this article as no new data were created or analyzed in this study.
