# Supplementary material for: Strategic roadmap to assess forest vulnerability under air pollution and climate change
Source: Glob Chang Biol. 2022 Jun 21;28(17):5062–85. doi: 10.1111/gcb.16278 (PMC9541114; doi:10.1111/gcb.16278)
Supplement: Supplementary file 1 — Appendix S1. [file GCB-28-5062-s001.doc]

Supplementary materials

**Analysis of publications pertinent to the research topic**

The research topic was defined as “forest vulnerability under air pollution and climate change”. ISI Web of Science database was searched on 05 June 2020 for publications, the title of which included (climat* and pollut*) and (tree* or forest*). The publications found by this search were classified as follows:


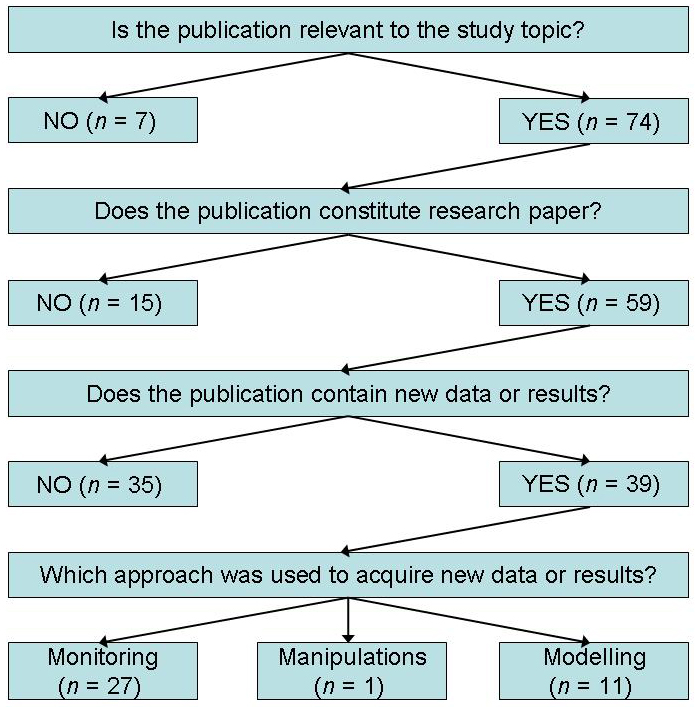


**Identified publications**

*1. Publications which are not relevant to the research topic*

Comrie AC. 1994. A synoptic climatology of rural ozone pollution at three forest sites in Pennsylvania. Atmospheric Environment 28, 1601-1614.

Davies SJ & Unam L. 1999. Smoke-haze from the 1997 Indonesian forest fires: effects on pollution levels local climate atmospheric CO_2_ concentrations and tree photosynthesis. Forest Ecology and Management 124, 137-144.

Fukuda K. 2007. Noise reduction approach for decision tree construction: a case study of knowledge discovery on climate and air pollution. 2007 IEEE symposium on computational intelligence and data mining, 697-704.

Kiss M, Takacs A, Pogacsas R & Gulyas A. 2015. The role of ecosystem services in climate and air quality in urban areas: Evaluating carbon sequestration and air pollution removal by street and park trees in Szeged (Hungary). Moravian Geographical Reports 23, 36-46.

Neal C, Robinson M, Reynolds B, Neal M, Rowland P, Grant S, Norris D, Williams B, Sleep D & Lawlor A. 2010. Hydrology and water quality of the headwaters of the River Severn: stream acidity recovery and interactions with plantation forestry under an improving pollution climate. Science of the Total Environment 408, 5035-5051.

Rodriguez-Germade I, Mohamed KJ, Rey D, Rubio B & Garcia A. 2014. The influence of weather and climate on the reliability of magnetic properties of tree leaves as proxies for air pollution monitoring. Science of the Total Environment 468, 892-902.

Skeffington RA & Hill TJ. 2012. The effects of a changing pollution climate on throughfall deposition and cycling in a forested area in southern England. Science of the Total Environment 434, 28-38.

*2. Publications which are relevant to the research topic*

*2.1. Information materials (prefaces, editorials, reviews etc.)*

Abrams MD. 2011. Editorial: Adaptations of forest ecosystems to air pollution and climate change. Tree Physiology 31, 258-261.

Augustaitis A, Bytnerowicz A & Paoletti E. 2014. Biological reactions of forests to climate change and air pollution. Environmental Pollution 184, 657-658.

Barth H. 1969. Air pollution and urban climate in industrial zones of Rheinland-Westphalia and effect on lichen growth with respect to trees - German - Domrös M. Geographische Zeitschrift 57, 221-222.

Bytnerowicz A. 1997. Air pollution and climate change effects on forests in central and eastern Europe. Environmental Pollution, 98, 271-271.

Bytnerowicz A, Arbaugh M, Fenn M, Gimeno BS & Paoletti E. 2008. Introduction: Forests under anthropogenic pressure - Effects of air pollution climate change and urban development. Environmental Pollution 155, 389-390.

Huttunen S. 2005. Forests under changing climate, enhanced UV and air pollution. Introductory remarks to the Special Issue. Environmental Pollution 137, 371-371.

Izuta T. 2019. Actions for sustainable forest ecosystems under air pollution and climate change : Preface. Journal of Agricultural Meteorology 75, 1-2.

Paoletti E & Serengil Y. 2011. Preface to the IUFRO special section "Adaptation of forest ecosystems to air pollution and climate change". Environmental Pollution 159, 1023-1023.

Paoletti E & Sicard P. 2016. Preface to the IUFRO RG7.01 special section "Global Challenges of Air Pollution and Climate Change to Forests". Environmental Pollution 213, 975-976

Paoletti E & Tuovinen JP. 2011. COST Action FP0903: "Research monitoring and modelling in the study of climate change and air pollution impacts on forest ecosystems". iForest-Biogeosciences and Forestry 4, 160-161.

Paoletti E, Bytnerowicz A & Schaub M. 2007. Key studies on air pollution and climate change impacts on forests: an introduction. Environmental Monitoring and Assessment 128, 1-3.

Ritter W. 1966. Air pollution and climate in the industrialized part of Rheinland-Westfalen and its effect on lichen growing on trees - German – Domrös M. Mitteilungen der Osterreichischen Geographischen Gesellschaft 108, 384-385.

Schaub M & Paoletti E. 2007. Introductory remarks to the special issue: XXII IUFRO World Congress 2005 Brisbane. Air pollution and climate change: A global overview of the effects on forest vegetation. Environmental Pollution 147, 429-429.

Schaub M, Matyssek R & Wieser G. 2010. Preface to the special section of the IUFRO conference on air pollution and climate change effects on forest ecosystems. Environmental Pollution 158, 1985-1985.

Vaughan A. 2020. Make polluters pay for new trees. New Scientist 245, 15-15.

*2.2. Research papers*

*2.2.1. Research papers which do not contain new data or results*

Bytnerowicz A, Fenn M, McNulty S, Yuan FM, Pourmokhtarian A, Driscoll C & Meixner T. 2013. Interactive effects of air pollution and climate change on forest ecosystems in the United States: current understanding and future scenarios. Developments in Environmental Science 13, 333-369.

Bytnerowicz A, Omasa K & Paoletti E. 2005. Integrated effects of air pollution and climate change on forests: a northern hemisphere perspective. Environmental Pollution 147, 438-445.

Clarke N, Fischer R, de Vries W, Lundin L, Papale D, Vesala T, Merila P, Matteucci G, Mirtl M, Simpson D & Paoletti E. 2011. Availability accessibility quality and comparability of monitoring data for European forests for use in air pollution and climate change science. iForest-Biogeosciences and Forestry 4, 162-166.

Dixon RK & Wisniewski J. 1995. Global forest systems: an uncertain response to atmospheric pollutants and global climate change? Water Air And Soil Pollution 85, 101-110.

Kuiper PJC. 1998. Cost 614: Impacts of elevated CO_2_ air pollutants and climate change on tree physiology (ICAT): Review of a cost action - (December 1991-December 1996). Forestry Sciences 52, 365-368.

Laitat E & Loosveldt P. 1992. Open-top chambers for study of the physiology of acclimated trees under enhanced CO_2_ in natural pollution climate. Responses of forest ecosystems to environmental changes, 653-654.

Matyssek R, Clarke N, Cudlin P, Mikkelsen TN, Tuovinen JP, Wieser G & Paoletti E. 2013. Climate change, air pollution and global challenges: understanding and perspectives from forest research. Developments in Environmental Science 13, 3-16.

Matyssek R, Kozovits AR, Schnitzler JP, Pretzsch H, Dieler J & Wieser G. 2014. Forest trees under air pollution as a factor of climate change. Plant Ecophysiology, 9, 117-163.

Matyssek R, Kozovits AR, Wieser G, Augustaitiene I & Augustaitis A. 2014. Biological reactions of forests to climate change and air pollution. European Journal of Forest Research 133, 671-673.

Matyssek R, Schaub M & Wieser G. 2010. Air pollution and climate change effects on forest ecosystems: new evidence. European Journal of Forest Research 129, 417-419.

Matyssek R, Wieser G, Calfapietra C, de Vries W, Dizengremel P, Ernst D, Jolivet Y, Mikkelsen TN, Mohren GMJ, Le Thiec D, Tuovinen JP, Weatherall A & Paoletti E. 2012. Forests under climate change and air pollution: Gaps in understanding and future directions for research. Environmental Pollution 160, 57-65.

McLaughlin S & Percy K. 1999. Forest health in North America: Some perspectives on actual and potential roles of climate and air pollution. Water Air and Soil Pollution 116, 151-197.

Mickler RA, McNulty SG, Birdsey RA & Hom J. 2003. Responses of forests in the eastern US to air pollution and climate change. Developments in Environmental Science 3, 345-358.

Paoletti E, Bytnerowicz A, Andersen C, Augustaitis A, Ferretti M, Grulke N, Gunthardt-Goerg MS, Innes J, Johnson D, Karnosky D, Luangjame J, Matyssek R, McNulty S, Muller-Starck G, Musselman R & Percy K. 2007. Impacts of air pollution and climate change on forest ecosystems - Emerging research needs. TheScientificWorldJournal 7, 1-8.

Paoletti E, de Vries W, Mikkelsen TN, Ibrom A, Larsen KS, Tuovinen JP, Serengil Y, Yurtseven I, Wieser G & Matyssek R. 2013. Key indicators of air pollution and climate change impacts at forest supersites. Developments in Environmental Science 13, 497-518.

Payer Hd, Blank Lw, Bosch C, Gnatz G, Schmolke W & Schramel P. 1986. Simultaneous exposure of forest trees to pollutants and climatic stress. Water Air And Soil Pollution 31, 485-491.

Pregitzer KS, Reed DD, Mroz GD, Burton AJ, Witter JA & Zak DA. 1996. Climatic and pollution influences on ecosystem processes in northern hardwood forests. USDA Forest Service General Technical Report 214, 83-88.

Serengil Y, Augustaitis A, Bytnerowicz A, Grulke N, Kozovitz AR, Matyssek R, Muller-Starck G, Schaub M, Wieser G, Coskun AA & Paoletti E. 2011. Adaptation of forest ecosystems to air pollution and climate change: a global assessment on research priorities. iForest-Biogeosciences and Forestry 4, 44-48.

Sicard P, Augustaitis A, Belyazid S, Calfapietra C, de Marco A, Fenn M, Bytnerowicz A, Grulke N, He S, Matyssek R, Serengil Y, Wieser G & Paoletti E. 2016. Global topics and novel approaches in the study of air pollution climate change and forest ecosystems. Environmental Pollution, 213, 977-987.

Unsworth MH. 1992. Climate and air pollution as determinants of forest ecosystem processes. Responses of forest ecosystems to environmental changes, 63-75.

*2.2.2. Research papers which contain new data or results*

*2.2.2.1. Observational (monitoring) studies*

Aguiar-Silva C, Brandao SE, Domingos M & Bulbovas P. 2016. Antioxidant responses of Atlantic Forest native tree species as indicators of increasing tolerance to oxidative stress when they are exposed to air pollutants and seasonal tropical climate. Ecological Indicators 63, 154-164.

Ashby WC. 1972. Tree growth air pollution and climate near Laporte Ind. Bulletin of the American Meteorological Society 53, 246-251.

Beck W. 2009. Growth patterns of forest stands - the response towards pollutants and climatic impact. iForest-Biogeosciences and Forestry 2, 4-6.

Bo M, Mercalli L, Pognant F, Berro DC & Clerico M. 2020. Urban air pollution, climate change and wildfires: The case study of an extended forest fire episode in northern Italy favoured by drought and warm weather conditions. Energy Reports 6, 781-786.

Breymeyer A. 1997. Transect studies of pine forests along parallel 52°N, 12-32°E and along a pollution gradient in central Europe: general assumptions climatic conditions and pollution deposition. Environmental Pollution 98, 335-345.

Burton AJ, Pregitzer KS & Macdonald NW. 1993. Foliar nutrients in sugar maple forests along a regional pollution-climate gradient. Soil Science Society of America Journal 57, 1619-1628.

Coxson D, Bjork C & Bourassa MD. 2014. The influence of regional gradients in climate and air pollution on epiphytes in riparian forest galleries of the upper Fraser River watershed. Botany 92, 23-45.

Vacek S, Vacek Z, Remes J, Bilek L, Hunova I, Bulusek D, Putalova T, Kral J & Simon J. 2017. Sensitivity of unmanaged relict pine forest in the Czech Republic to climate change and air pollution. Trees-Structure and Function 31, 1599-1617.

Cudlin P, Sejak J, Pokorny J, Albrechtova J, Bastian O & Marek M. 2013. Forest ecosystem services under climate change and air pollution. Developments in Environmental Science 13, 521-546.

Fleischer P, Godzik B, Bicarova S & Bytnerowicz A. 2005. Effects of air pollution and climate change on forests of the Tatra Mountains, Central Europe. Plant responses to air pollution and global change, 111-121.

Godzik S, Staszewski T & Szdzuj J. 1995. Deposition of air pollutants to forest ecosystems along pollution and climatic gradients in Poland. Studies In Environmental Science 64 425-426.

Kantarci MD. 1997. The effects of air pollution and the combination of species in the forests of the mountainous areas of Marmara region based on the vertical climate zones. Air quality management: at urban regional and global scales. 10th Regional IUAPPA Conference, 511-518.

Kantarci MD. 2011. Vertical climate zones in Biga peninsula: the impact of climate change and air pollution on forests. Procedia—Social and Behavioral Sciences 19, 797-810.

Kralicek I, Vacek Z, Vacek S, Remes J, Bulusek D, Kral J, Stefancik I & Putalova T. 2017. Dynamics and structure of mountain autochthonous spruce-beech forests: impact of hilltop phenomenon air pollutants and climate. Dendrobiology 77, 119-137.

Leonelli G, Battipaglia G, Siegwolf RTW, Saurer M, di Cella UM, Cherubini P & Pelfini, M. 2012. Climatic isotope signals in tree rings masked by air pollution: A case study conducted along the Mont Blanc Tunnel access road (Western Alps, Italy). Atmospheric Environment 61, 169-179.

Locosselli GM, de Camargo EP, Moreira TCL, Todesco E, Andrade MD, de Andre CDS, de Andre PA, Singer JM, Ferreira LS, Saldiva PHN, Buckeridge MS. 2019. The role of air pollution and climate on the growth of urban trees. Science of the Total Environment 666, 652-661.

Mathias JM & Thomas RB. 2018. Disentangling the effects of acidic air pollution, atmospheric CO_2_, and climate change on recent growth of red spruce trees in the Central Appalachian Mountains. Global Change Biology 24, 3938-3953.

Mcclenahen JR & Dochinger LS. 1985. Tree-ring response of white oak to climate and air-pollution near the Ohio river valley. Journal of Environmental Quality 14, 274-280.

Mikulenka P, Prokupkova A, Vacek Z, Vacek S, Bulusek D, Simon J, Simunek V & Hajek V. 2020. Effect of climate and air pollution on radial growth of mixed forests: *Abies alba* Mill. vs. *Picea abies* (L.) Karst. Central European Forestry Journal 66, 23-36.

Miller DE & Watmough SA. 2009. Air pollution, climate, soil acidity and indicators of forest health in Ontario's sugar maple forests. Canadian Journal of Forest Research 39, 2065- 2079.

Pivoras A, Mikalajunas M, Juonyte D & Pivoras G. 2017. Integrated effect of climate and air pollutants on diurnal tree ring formation of Scots pine, Norway spruce and silver and downy birch trees stem circumference. 8th international scientific conference rural development 2017: bioeconomy challenges, 751-756.

Polle A, Mossnang M, Vonschonborn A, Sladkovic R & Rennenberg H. 1992. Field studies on Norway spruce trees at high altitudes. 1. Mineral pigment and soluble protein contents of needles as affected by climate and pollution. New Phytologist 121, 89-99.

Putalova T, Vacek Z, Vacek S, Stefancik I, Bulusek D & Kral J. 2019. Tree-ring widths as an indicator of air pollution stress and climate conditions in different Norway spruce forest stands in the Krkonose Mts. Central European Forestry Journal 65, 21-33.

Staszewski T, Godzik S, Kubiesa P & Szdzuj J. 1999. Fate of nitrogen compounds deposited to spruce (*Picea abies* Karst.) and pine (*Pinus silvestris* L.) forests located in different air pollution and climatic conditions. Water Air and Soil Pollution 116, 121-127.

Staszewski T, Lukasik W, Godzik S, Szdzuj J & Uzieblo AK. 1998. Climatic and air pollution gradient studies on coniferous trees health status needles wettability and chemical characteristics. Chemosphere 36, 901-905.

Swierkosz K, Reczynska K & Pech P. 2014. Is the plant species composition of Silver fir mixed forest in the Polish highlands affected by air pollution and climate warming? Phytocoenologia 44, 45-53.

Ulrich B. 1984. Acid rain and forest demise - the overlap of natural climatic stress with air pollution. Umschau 84, 348-355.

Vacek S, Hunova I, Vacek Z, Hejcmanova P, Podrazsky V, Kral J, Putalova T & Moser WK. 2015. Effects of air pollution and climatic factors on Norway spruce forests in the Orlick, hory Mts. (Czech Republic), 1979-2014. European Journal of Forest Research 134, 1127-1142.

*2.2.2. Experimental (manipulative) studies*

Furlan CM, Domingos M & Salatino A. 2007. Effects of initial climatic conditions on growth and accumulation of fluoride and nitrogen in leaves of two tropical tree species exposed to industrial air pollution. Science of the Total Environment 374, 399-407.

*2.2.1. Modelling studies*

Belyazid S, Phelan J, Nihlgard B, Sverdrup H, Driscoll C, Fernandez I, Aherne J, Teeling-Adams LM, Bailey S, Arsenault M, Cleavitt N, Engstrom B, Dennis R, Sperduto D, Werier D & Clark C. 2019. Assessing the effects of climate change and air pollution on soil properties and plant diversity in northeastern US hardwood forests: model setup and evaluation. Water Air and Soil Pollution 230, 106.

Dirnbock T, Djukic I, Kitzler B, Kobler J, Mol-Dijkstra JP, Posch M, Reinds GJ, Schlutow A, Starlinger F & Wamelink WGW. 2017. Climate and air pollution impacts on habitat suitability of Austrian forest ecosystems. PLoS ONE 12, e0184194.

Kremer RG. 1991. Simulating forest response to air pollution – integrating physiological responses to sulfur dioxide with climate-dependent growth processes. Ecological Modelling 54, 111-126.

McDonnell TC, Reinds GJ, Sullivan TJ, Clark CM, Bonten LTC, Mol-Dijkstra JP, Wamelink GWW & Dovciak M. 2018. Feasibility of coupled empirical and dynamic modeling to assess climate change and air pollution impacts on temperate forest vegetation of the eastern United States. Environmental Pollution 234, 902-914.

Nizzetto L & Perlinger JA. 2012. Climatic, biological, and land cover controls on the exchange of gas-phase semivolatile chemical pollutants between forest canopies and the atmosphere. Environmental Science & Technology 46, 2699-2707.

Park MJ, Park JY, Shin HJ, Lee MS, Park GA, Jung IK & Kim SJ. 2010. Projection of future climate change impacts on nonpoint source pollution loads for a forest dominant dam watershed by reflecting future vegetation canopy in a Soil and Water Assessment Tool model. Water Science and Technology 61, 1975-1986.

Phelan J, Belyazid S, Jones P, Cajka J, Buckley J & Clark C. 2016. Assessing the effects of climate change and air pollution on soil properties and plant diversity in sugar maple-beech-yellow birch hardwood forests in the Northeastern United States: model simulations from 1900 to 2100. Water Air and Soil Pollution 227, 84.

Wai KM, Tan TZ, Morakinyo TE, Chan TC & Lai A. 2020. Reduced effectiveness of tree planting on micro-climate cooling due to ozone pollution: a modeling study. Sustainable Cities and Society 52, 101803.

Verrico BM, Weiland J, Perkins TD, Beckage B & Keller SR. 2020. Long-term monitoring reveals forest tree community change driven by atmospheric sulphate pollution and contemporary climate change. Diversity and Distributions 26, 270-283.

Zoran MA, Zoran LFV & Dida AI. 2008. The assessment of air pollution and climatic changes impacts on mountain forest ecosystems by satellite remote sensing data. Remote Sensing for Agriculture, Ecosystems, and Hydrology: Proceedings of SPIE, 7104, UNSP 71040Z.

Tuovinen JP, Hakola H, Karlsson PE & Simpson D. 2013. Air pollution risks to Northern European forests in a changing climate. Developments in Environmental Science 13, 77-99.
